# Molecular classification and biomarkers of outcome with immunotherapy in extensive-stage small-cell lung cancer: analyses of the CASPIAN phase 3 study

**DOI:** 10.1186/s12943-024-02014-x

**Published:** 2024-05-30

**Authors:** Mingchao Xie, Miljenka Vuko, Jaime Rodriguez-Canales, Johannes Zimmermann, Markus Schick, Cathy O’Brien, Luis Paz-Ares, Jonathan W. Goldman, Marina Chiara Garassino, Carl M. Gay, John V. Heymach, Haiyi Jiang, J. Carl Barrett, Ross A. Stewart, Zhongwu Lai, Lauren A. Byers, Charles M. Rudin, Yashaswi Shrestha

**Affiliations:** 1 Oncology Data Science, AstraZeneca, Waltham, MA USA; 2 Computational Pathology, AstraZeneca, Munich, Germany; 3 Translational Medicine, Oncology R&D, AstraZeneca, Gaithersburg, MD USA; 4 Biostatistics, Oncology R&D, AstraZeneca, Cambridge, UK; 5 Department of Medical Oncology, Hospital Universitario 12 de Octubre, Madrid, Spain; 6 David Geffen School of Medicine at UCLA, Los Angeles, CA USA; 7 Fondazione IRCCS Istituto Nazionale dei Tumori, Milan, Italy; 8 The University of Chicago, Chicago, IL USA; 9 The University of Texas MD Anderson Cancer Center, Houston, TX USA; 10 Oncology R&D, AstraZeneca, Gaithersburg, MD USA; 11 Translational Medicine, AstraZeneca, Waltham, MA, United States; 12 Translational Medicine, Oncology R&D, AstraZeneca, Cambridge, UK; 13 Memorial Sloan Kettering Cancer Center, New York, NY USA

**Keywords:** Antigen presentation machinery, Biomarkers, CTLA-4, Gene expression profiling, Molecular subtyping, PD-L1, SCLC subtypes, Small-cell lung cancer, T-cell inflamed signature

## Abstract

**Background:**

We explored potential predictive biomarkers of immunotherapy response in patients with extensive-stage small-cell lung cancer (ES-SCLC) treated with durvalumab (D) + tremelimumab (T) + etoposide-platinum (EP), D + EP, or EP in the randomized phase 3 CASPIAN trial.

**Methods:**

805 treatment-naïve patients with ES-SCLC were randomized (1:1:1) to receive D + T + EP, D + EP, or EP. The primary endpoint was overall survival (OS). Patients were required to provide an archived tumor tissue block (or ≥ 15 newly cut unstained slides) at screening, if these samples existed. After assessment for programmed cell death ligand-1 expression and tissue tumor mutational burden, residual tissue was used for additional molecular profiling including by RNA sequencing and immunohistochemistry.

**Results:**

In 182 patients with transcriptional molecular subtyping, OS with D ± T + EP was numerically highest in the SCLC-inflamed subtype (*n* = 10, median 24.0 months). Patients derived benefit from immunotherapy across subtypes; thus, additional biomarkers were investigated. OS benefit with D ± T + EP versus EP was greater with high versus low *CD8A* expression/CD8 cell density by immunohistochemistry, but with no additional benefit with D + T + EP versus D + EP. OS benefit with D + T + EP versus D + EP was associated with high expression of *CD4* (median 25.9 vs. 11.4 months) and antigen-presenting and processing machinery (25.9 vs. 14.6 months) and MHC I and II (23.6 vs. 17.3 months) gene signatures, and with higher MHC I expression by immunohistochemistry.

**Conclusions:**

These findings demonstrate the tumor microenvironment is important in mediating better outcomes with D ± T + EP in ES-SCLC, with canonical immune markers associated with hypothesized immunotherapy mechanisms of action defining patient subsets that respond to D ± T.

**Trial registration:**

ClinicalTrials.gov, NCT03043872.

**Supplementary Information:**

The online version contains supplementary material available at 10.1186/s12943-024-02014-x.

## Introduction

Small-cell lung cancer (SCLC) is an aggressive type of cancer comprising approximately 15% of all lung cancer cases that is associated with a particularly poor prognosis [1]. SCLC has been shown to have a high prevalence of *RB1* and *TP53* inactivation [2, 3]; however, a lack of further biological understanding of the disease has restricted biomarker development until recently [4–7]. Consequently, even though only a minority of patients exhibit long-term survival benefit, the treatment of extensive-stage (ES) SCLC currently uses an all-comers approach [8], as limited efficacious molecularly targeted therapies or treatment options are available [1].

Combination therapy with the anti-programmed cell death ligand-1 (PD-L1) monoclonal antibody durvalumab (D) and etoposide plus cisplatin or carboplatin (EP) is a first-line standard of care for ES-SCLC [8] based on the results of the randomized, open-label, phase 3 CASPIAN trial (NCT03043872) [9, 10]. In CASPIAN, treatment-naïve patients with ES-SCLC received EP alone, D + EP, or D + EP and the anti-cytotoxic T-lymphocyte-associated antigen 4 (CTLA-4) monoclonal antibody tremelimumab (D + T + EP) [9, 10]. D + EP demonstrated a significant overall survival (OS) benefit compared with EP [9], which was maintained after a median follow-up of > 3 years (hazard ratio [HR] = 0.71; 95% confidence interval [CI], 0.60–0.86) [11]. D + T + EP showed numerical improvement in OS versus EP at this analysis time-point (HR = 0.81; 95% CI, 0.67–0.97) [11], with 16% and 14% of patients being long-term survivors (alive after a median follow-up of 39.4 months) in the D + EP and D + T + EP arms, respectively, compared with 5% in the EP arm [11].

Comprehensive molecular characterization of SCLC is important for providing a better understanding of disease heterogeneity and may consequently lead to identification of predictive biomarkers for current and future treatment options, including immune checkpoint inhibitor (ICI)-based therapy [5, 12]. However, while PD-L1 expression and tumor mutational burden (TMB) have been identified as predictive biomarkers for outcomes with ICIs in other indications, including non-small-cell lung cancer (NSCLC) [13, 14] and metastatic triple-negative breast cancer [15], there is no clear evidence from randomized phase 3 studies that PD-L1 expression or TMB predicts outcomes with immunochemotherapy in ES-SCLC. Analyses of the CASPIAN trial [16], the IMpower133 trial of atezolizumab plus EP versus EP [17, 18], and the KEYNOTE-604 trial of pembrolizumab plus EP versus EP [19] showed that tissue TMB (tTMB) was not associated with outcomes with immunotherapy. Similarly, in CASPIAN [16], IMpower133 [17, 18], and KEYNOTE-604 [20], PD-L1 expression was not associated with outcomes to anti-PD-(L)1 antibody plus EP therapy, although there was some suggestion from an exploratory analysis in CASPIAN that PD-L1 expression might predict OS benefit with D + T + EP versus EP [16].

Thus, there is a need to explore other molecular biomarkers in ES-SCLC to identify patients most likely to benefit from ICI therapy. In the past 5 years, there have been substantial developments in identifying distinct molecular subtypes of SCLC based on differential expression of transcription factor genes (*ASCL1*, *NEUROD1*, *POU2F3* – respectively, SCLC-A, SCLC-N, and SCLC-P subtypes) or low/lack of expression of these genes. In initial work, expression of the transcriptional regulator *YAP1* (SCLC-Y subtype) [4, 6, 21] was also proposed as a potential biomarker. Subsequently, a subset of ~ 15–20% of SCLC tumors were found to have an immunologically ‘inflamed’ gene expression pattern (SCLC-I subtype), which was associated with benefit from the addition of immunotherapy (atezolizumab) to EP [5, 21, 22]. An 18-gene T-cell inflamed gene expression signature (enriched in SCLC-I) [5] has been derived based on data on clinical benefit with the PD-1 antibody pembrolizumab that contains interferon-γ-responsive genes related to antigen presentation, chemokine expression, cytotoxic activity, and adaptive immune resistance [19, 23]. The specific molecular subtypes of SCLC have been further characterized by association with expression of neuroendocrine markers and limited expression of immune-associated genes and/or an antigen-presenting and processing machinery (APM) signature in the immune-cold SCLC-A and SCLC-N subtypes, in contrast to the elevated expression of immune checkpoint molecules and human leukocyte antigens (HLAs) seen in the immunologically inflamed SCLC-I subtype [1, 24]. Consistent with this, Mahadevan et al. described an SCLC subtype with non-neuroendocrine features that is associated with high major histocompatibility complex (MHC) class I expression and responsiveness to immunotherapy [25].

Here, we present exploratory analyses from the CASPIAN trial that provide further insights into the heterogeneity of SCLC subtypes. In particular, these analyses focus on identifying potential biomarkers that may predict clinical benefit with immunotherapy, including biomarkers that may predict responsiveness to durvalumab and that can identify patients who may benefit from the addition of tremelimumab to D + EP.

## Methods

### CASPIAN study design

The phase III CASPIAN trial was a randomized, open-label, sponsor-blind trial in which 805 treatment-naïve patients aged ≥ 18 years (≥ 20 years in Japan) with histologically or cytologically confirmed ES-SCLC from 23 countries in Europe, Asia, and North and South America were randomly assigned in a 1:1:1 ratio to receive treatment with D + T + EP, D + EP, or EP alone, as previously reported [9–11]. Patients required a World Health Organization (WHO) performance status of 0 or 1, measurable disease per Response Evaluation Criteria in Solid Tumors (RECIST) version 1.1, life expectancy of ≥ 12 weeks from the start of the study, and body weight ≥ 30 kg, and they had to be eligible for first-line platinum-based chemotherapy [9]. Patients were required to provide an archived tumor tissue block (or ≥ 15 newly cut unstained slides) at screening, if these samples existed [9]. The study was conducted in accordance with the International Conference on Harmonisation good clinical practice guidelines, the Declaration of Helsinki, and applicable local regulations. All patients provided written informed consent prior to study participation. The study protocol and all modifications was approved by the independent ethics committees or institutional review boards, and by the relevant regulatory authorities, for all 209 study sites [9].

The exploratory objectives of CASPIAN included investigation of the relationships between efficacy outcomes and: PD-L1 expression and distribution in the tumor microenvironment (TME); TMB and/or somatic mutations/genomic alterations; gene expression of select genes within the TME; and delta-like ligand 3 (DLL3) expression. A further exploratory objective was to explore potential biomarkers in tumor and blood that may influence disease progression and/or prospectively identify patients likely to respond to D-based or D + T-based treatment.

### RNAseq process and analysis

RNA extraction and whole-transcriptome sequencing (WTS) library preparation were performed as described in Additional File 1, Supplementary Methods. The RNA sequencing (RNAseq) pipeline implemented in bcbio-nextgen (version 1.2.7) was used for quality control and gene expression quantification (Additional File 1, Supplementary Methods). Protein-coding genes with a Transcripts Per Million value exceeding 0.5 in more than 25% of samples were selected for downstream analysis. High expression was characterized as the top quartile of patients exhibiting the highest expression and low expression as the other three quartiles. Cox regression was employed to identify the genes associated with clinical outcome with each treatment, considering a HR of < 1 and a p-value of < 0.05. Pathway enrichment score was calculated by Fisher exact test against the Kyoto Encyclopedia of Genes and Genomes (KEGG) pathway derived from Molecular Signatures Database (MSigDB). Gene Set Enrichment Analysis (GSEA) plot was conducted by R package (fgsea v1.22.0). APM and MHC-I signature were derived from KEGG/MSigDB (https://www.gsea-msigdb.org/gsea/msigdb/cards/KEGG_ANTIGEN_PROCESSING_AND_PRESENTATION) and Rooney et al. [26], respectively. Gene signature score was defined as the average expression value of the genes in the signature.

TMB was assessed in tissue biopsy samples using the FoundationOne® CDx targeted panel assay (Foundation Medicine, Cambridge, MA) per the previously described algorithm [27]. tTMB was calculated based on the sum of all synonymous and non-synonymous base substitutions and short insertions/deletions in the coding region from the FoundationOne® CDx next-generation sequencing (NGS) assay, after removing germline and oncogenic driver mutations. SCLC subtyping was defined per the method of Gay et al. [5] (SCLC-A, SCLC-N, SCLC-P, and SCLC-I subtypes) and also per the method of Rudin et al. [6] (SCLC-A, SCLC-N, SCLC-P, and SCLC-Y subtypes), and the 18-gene T-cell inflamed gene expression signature was evaluated and the T-cell inflamed signature score calculated for each sample as previously described [22, 23] (Additional File 1, Supplementary Methods).

### PD-L1 immunohistochemistry

The VENTANA SP263 immunohistochemistry (IHC) assay (Ventana Medical Systems, Tucson, AZ) was used to assess expression of PD-L1 on tumor cells (TC) and immune cells (IC) [16], with expression status based on the percentage of cells with PD-L1 staining intensity above background. Testing was done in a central laboratory by pathologists trained and qualified by Ventana to score the samples at specific cut-offs.

### CD8 IHC

IHC was performed using an automated Ventana Discovery Ultra IHC staining platform (Roche, Ventana, Indianapolis, US). Following antigen retrieval using Tris/Borate/EDTA buffer solution (Roche, Ventana, cat 6,414,575,001) for 64 min at 96 °C, primary CD8 antibody (Dako, clone C8-144B, cat M710301-2) was incubated for 28 min at a dilution of 1/10 diluted in Dako antibody diluent with background reducing agents (Agilent, cat S3022). CD8-antibody-specific binding was detected using Omni-Map anti-Mouse HRP antibody according to the manufacturer’s instructions (Roche, Ventana, cat 5,269,652,001). Following cover slipping with DPX mounting media, slides were digitally scanned using a Leica Aperio Scanscope AT2 pathology slide scanner (Leica, Milton Keynes, UK) and provided to the project pathologist. Image analysis was performed as described in Additional File 1, Supplementary Methods.

### MHC I IHC

IHC was performed using an automated Leica Bond III IHC staining platform (Leica, Milton Keynes, UK). Following baking at 40 °C for 2 h and dewaxing for 30 min at 72 °C using Bond Dewax Solution (Leica, cat AR9222), the antigen retrieval step was conducted using EDTA-based pH 9 epitope retrieval solution for 30 min at 100 °C (Leica, cat AR9640). Tissue slides were incubated with Dako protein block serum free (Agilent, Z0909) for 10 min at ambient temperature. Primary HLA Class I ABC (MHC1) antibody (Abcam, cat ab70328) was incubated for 15 min at ambient temperature at a concentration of 0.1 µg/ml diluted in BOND Primary Antibody Diluent (Leica, cat AR9352). MHC1 antibody specific binding was detected using the Bond Polymer Refine detection kit according to the manufacturer’s instructions (Leica, cat DS9800). Following cover slipping with DPX mounting media, slides were digitally scanned using a Leica Aperio Scanscope AT2 pathology slide scanner (Leica, Milton Keynes, UK) and provided to the study pathologist. Scanned slides stained with MHC I were evaluated by the study pathologist who assigned an H-score of the tumor cells in each sample by multiplying the percentage of tumor cells positive for MHC I staining (0–100%) by the average intensity of staining (0 to 3+) of the tumor cells, with a final H-score range from 0 to 300 [25].

### Statistical analysis

The primary endpoint of CASPIAN was OS, defined as time from randomization to death from any cause. Progression-free survival (PFS, defined as time from randomization to objective disease progression or death from any cause in the absence of progression) was a secondary endpoint and was assessed by investigators per RECIST version 1.1. Median OS and PFS were estimated within all groups and subsets using Kaplan‒Meier methodology; HRs and 95% CIs were calculated using unstratified Cox proportional hazards models for all between-group comparisons for biomarkers of interest and for within-group comparisons according to biomarker status, except for comparisons with total summed group size of < 20 patients. The study was not designed or powered for formal statistical testing of OS and PFS between subgroups defined in these exploratory analyses.

## Results

### Study design and analysis populations

**Fig. 1:**
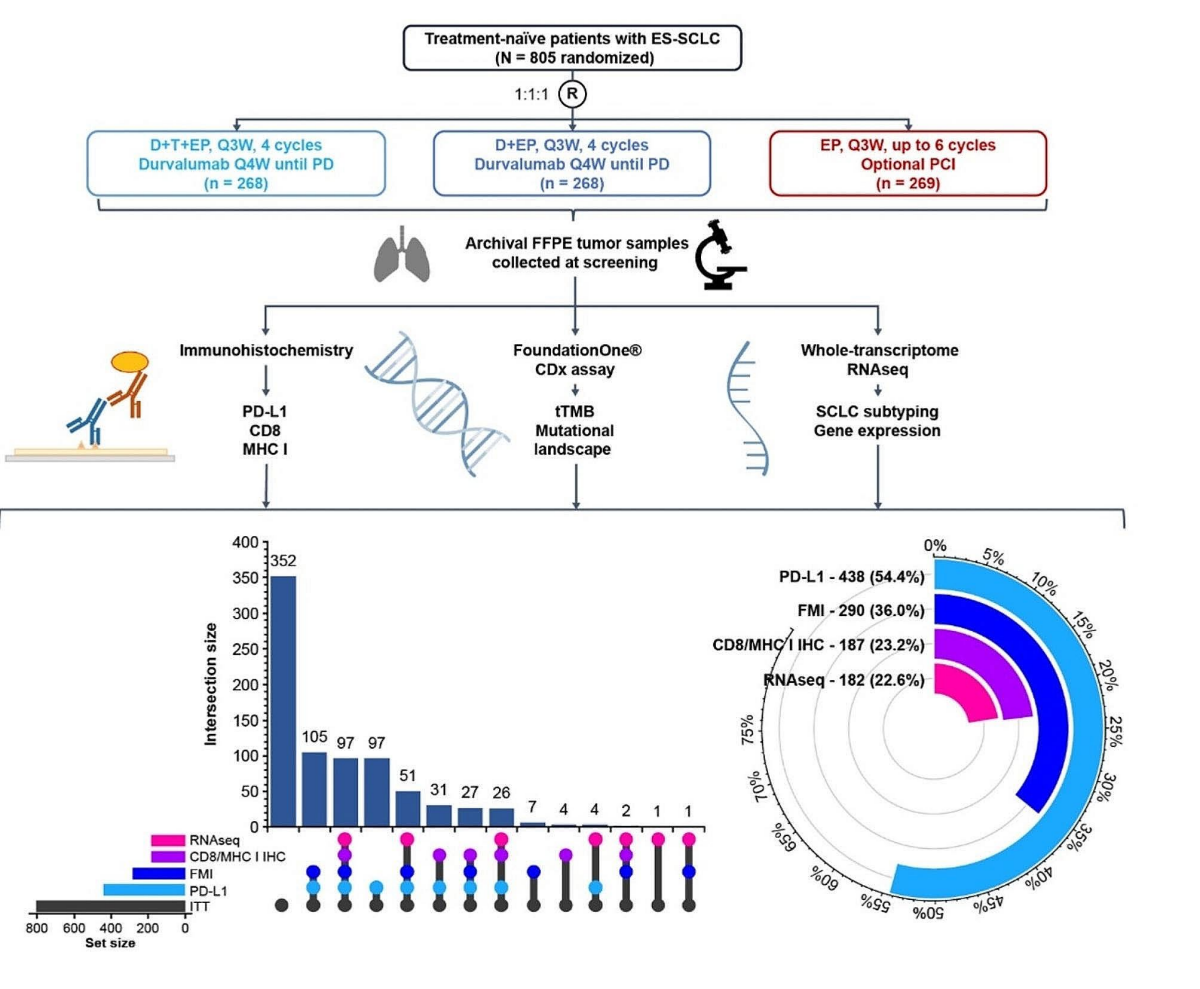
CASPIAN biomarker study design and molecular datasets. CASPIAN was a randomized phase 3 trial comparing D + T + EP, D + EP, and EP as first-line therapy in patients with ES-SCLC. Tissue-based analyses were conducted using archival tumor samples obtained from 65% of patients at screening

In the CASPIAN trial, 805 patients were randomized to receive D + T + EP (*n* = 268), D + EP (*n* = 268), or EP (*n* = 269). Tumor tissue samples were primarily assessed for PD-L1 expression and tTMB [16], and residual tissue was used for additional molecular profiling including RNAseq and IHC. For the purposes of these molecular biomarker analyses, we evaluated subgroups of 290 (36.0%), 182 (22.6%), and 187 (23.2%) patients for mutational landscape analysis, RNAseq, and IHC, respectively (Fig. 1). The RNAseq and IHC biomarker-evaluable populations (BEPs) were substantially overlapping.

The demographics and disease characteristics of the intent-to-treat (ITT) patient population in the D + T + EP, D + EP, and EP arms of the CASPIAN trial were reported previously [9, 10]. Key patient demographics and disease characteristics in the separate BEPs for these analyses, including the RNAseq BEP (*n* = 182), the CD8 IHC BEP (*n* = 169), and the MHC I IHC BEP (*n* = 175), showed that all BEPs were broadly representative of the ITT population (Table S1). The BEPs were slightly enriched for patients from the D + T + EP and D + EP arms (69.1–71.0%) compared with the ITT population (66.6%). Patient characteristics by treatment arm in the RNAseq (Table S2), CD8 IHC (Table S3), and MHC I IHC (Table S4) BEPs showed that these subsets were broadly comparable to each other. The percentage of patients with a WHO performance status of 1 was somewhat higher in the EP arm in the RNAseq BEP (75.9%) than in the ITT population (66.5%), and in the D + T + EP arm in the RNAseq (69.2%), CD8 (72.4%), and MHC I (72.9%) IHC BEPs compared to in the ITT population (59.3%). Treatment exposure was generally similar across the BEPs (Tables S2–4) and appeared somewhat greater in the BEPs than in the ITT population with, for example, 75.4–77.6% and 60.5%, respectively, receiving 5 doses of tremelimumab.

As reported previously, the median OS in ITT population with D + T + EP, D + EP, and EP was 10.4, 12.9, and 10.5 months, respectively, with HRs for the comparisons of D + T + EP and D + EP with EP of 0.81 and 0.72, respectively (Fig. S1A). As shown by the respective HRs, the OS benefit for D + T + EP and D + EP versus EP alone was greater in the RNAseq BEP (HRs 0.52 and 0.58; Fig. S1B) and the CD8 (HRs 0.55 and 0.49; Fig. S1C) and MHC I (HRs 0.57 and 0.48; Fig. S1D) IHC BEPs when compared to the ITT population, particularly for D + T + EP versus EP. Despite substantial overlaps between patients in the RNAseq and IHC BEPs, median OS values appeared numerically greater with D + T + EP and D + EP in the latter, potentially due to the medians being affected by a limited number of differing patients between the BEPs.

### Mutational landscape provides limited understanding of response to immunotherapy

**Fig. 2:**
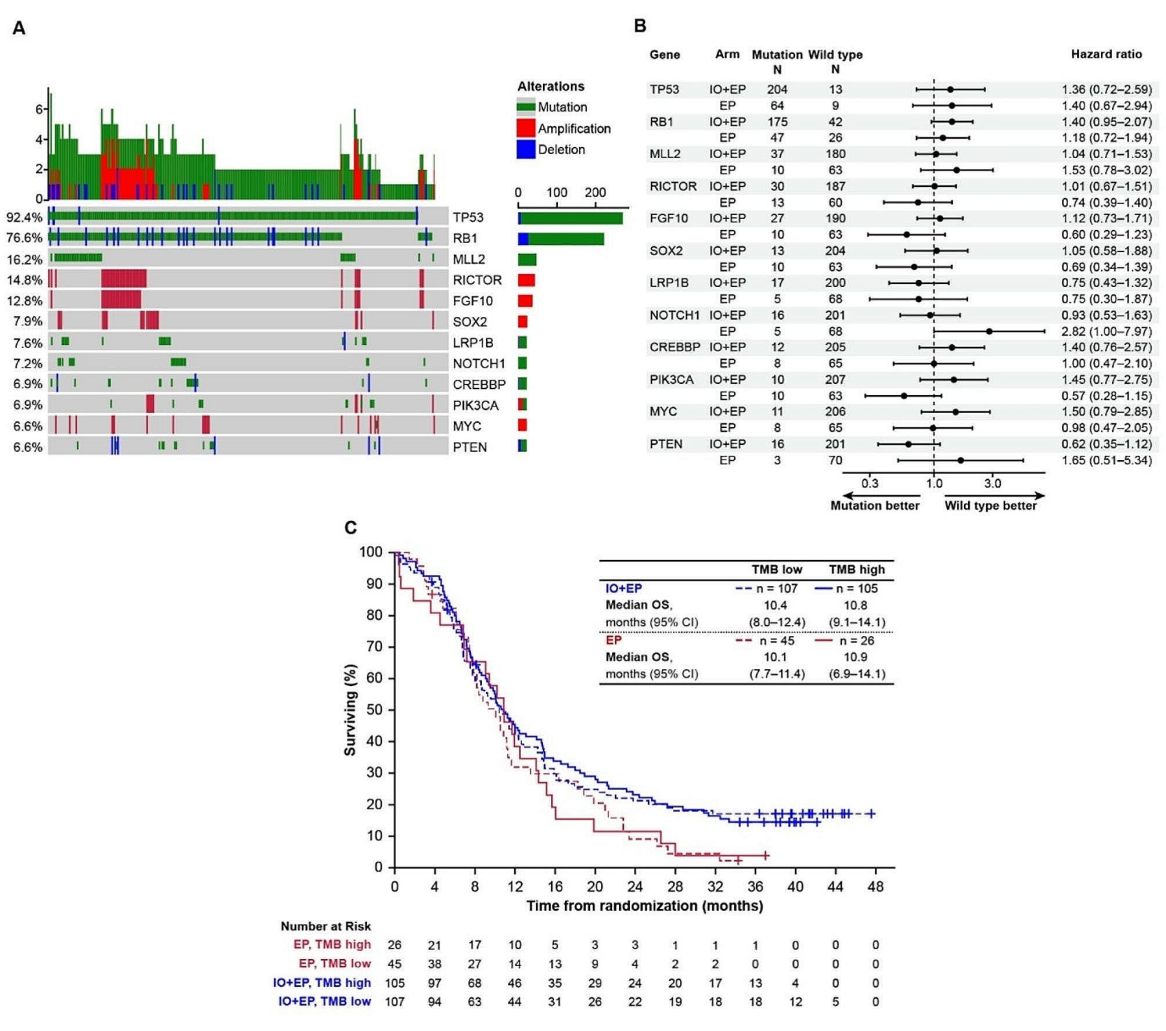
Common mutations in the CASPIAN population do not inform outcomes with immunotherapy. (**A**) Genes mutated in > 5% of patients in the CASPIAN FMI BEP (*n* = 290). (**B**) Association of mutation status of the most commonly mutated genes with OS (hazard ratio and 95% confidence interval, mutant versus wild-type) with immunotherapy (IO; D ± T) plus EP or EP alone. (**C**) Kaplan–Meier analyses of OS with IO + EP or EP in CASPIAN according to tTMB by 10 mut/Mb cut-off (D + T + EP group: median OS 9.1 [95% CI 6.9–11.4] and 10.0 [7.2–14.8] in the TMB low and TMB high cohorts, respectively; D + EP group, median OS 12.4 [95% CI 8.0–15.8] and 11.8 [8.6–14.9], respectively [16])

In our analysis of the mutational landscape in ES-SCLC patients in CASPIAN (*n* = 290), we found that the two most common mutations identified were of *TP53* in 268 (92.4%) and *RB1* in 222 (76.6%) patients, with all other gene mutations identified being seen in < 20% of patients (Fig. 2A). Mutational status of *TP53*, *RB1*, and other genes that were altered in ≥ 5% of patients was not associated with treatment response to D ± T + EP in CASPIAN (Fig. 2B), and mutational status of *TP53* and *RB1* did not inform outcomes in any of the treatment arms, including those containing immunotherapy (Fig. S2), with OS curves appearing broadly similar. We evaluated tTMB distribution in CASPIAN and compared this with data from two phase 3 studies in patients with metastatic NSCLC, MYSTIC [28] and NEPTUNE [29]. We found that the mean tTMB scores were comparable across the three studies, at 10.47, 10.04, and 10.45 mut/Mb, respectively (Fig. S3). Furthermore, as reported in a previous analysis of CASPIAN, we found no association between efficacy on D + T + EP or D + EP and tTMB status [16], and no association with OS using a cut-off of 10 mut/Mb in any of the treatment arms (Fig. 2C).

### Immune cells and immune markers in the tumor microenvironment associate with response to anti-PD-L1 immunotherapy

**Fig. 3:**
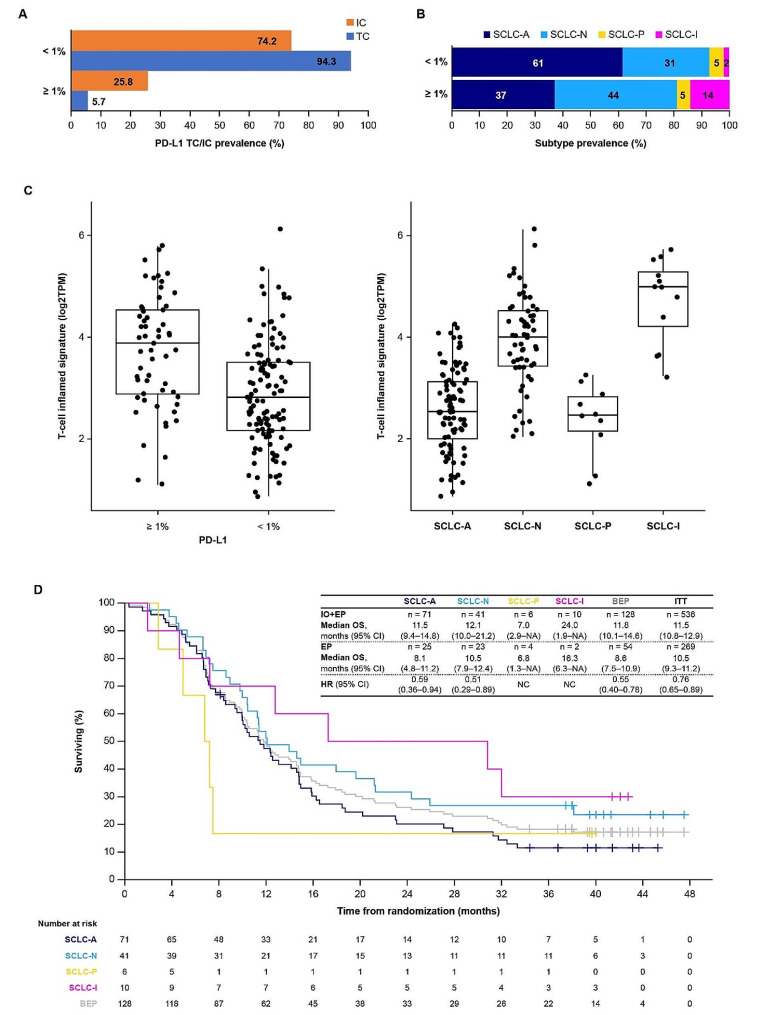
Immune phenotype and molecular subtyping, and association with OS. (**A**) Proportion of patients with PD-L1 expression of ≥ 1% or < 1% on TC or IC. (**B**) Patients grouped by PD-L1 expression of ≥ 1% or < 1% on TC and/or IC and categorized according to SCLC molecular subtype per the method of Gay et al. [5]. (**C**) T-cell inflamed signature in patients with PD-L1 TC/IC ≥ 1% versus < 1% and according to SCLC molecular subtype per the method of Gay et al. [5]. (**D**) OS by subtype, and median OS by subtype and treatment received (immunotherapy [IO; D ± T] plus EP or EP alone), as well as in the RNAseq BEP and ITT population, with HRs and 95% CIs showing relative OS benefit of IO + EP versus EP (for total group sizes > 20 patients). Median OS and progression-free survival values for each SCLC molecular subtype in each treatment group are shown in Table S5

A prior analysis from CASPIAN [16] demonstrated that the prevalence of PD-L1 expression on TC ≥ 1% was low in ES-SCLC, at 5.7%, but that the prevalence of PD-L1 expression on IC ≥ 1% was higher (25.8%) (Fig. 3A), resulting in an overall prevalence of PD-L1 TC and/or IC ≥ 1% of 28.3% [16]. This prior analysis showed no association of D + EP activity with PD-L1 TC/IC expression but a possible association of OS benefit with D + T + EP versus EP in the PD-L1 TC/IC ≥ 1% group [16]. We therefore built on PD-L1 IC expression by exploring immune contribution to response to immunotherapy. We investigated molecular subtype and T-cell inflamed signature score according to PD-L1 expression level (≥ 1% versus < 1%).

All SCLC molecular subtypes per the method of Gay et al. [5] (Fig. 3B) were shown to be present in both the PD-L1 TC/IC ≥ 1% and < 1% subgroups. The PD-L1 TC/IC ≥ 1% subgroup was enriched for the SCLC-I subtype (14% vs. 2% in the PD-L1 TC/IC < 1% subgroup), whereas the combined prevalence of the neuroendocrine subtypes SCLC-A and SCLC-N was lower in the PD-L1 TC/IC ≥ 1% subgroup versus the PD-L1 TC/IC < 1% subgroup (81% vs. 92%) (Fig. 3B). Additionally, we found that the mean 18-gene T-cell inflamed signature score was higher in the PD-L1 TC/IC ≥ 1% versus < 1% subgroup and in the SCLC-I subtype versus the other subtypes (Fig. 3C), consistent with previous findings [5].

Similarly, all SCLC molecular subtypes per the method of Rudin et al. [6] (Fig. S4A) were present in both the PD-L1 TC/IC ≥ 1% and < 1% subgroups, with a comparable relative pattern of subtype distribution. Per this classification method, the highest relative expression among four transcription factors determines the subtype, with *ASCL1* and *NEUROD1* subtypes classified as neuroendocrine and *POU2F3* and *YAP1* subtypes lacking neuroendocrine markers. Note that while *YAP1* RNA appears to be somewhat enriched in a putative subtype distinct from those with dominant expression of *ASCL1*, *NEUROD1*, and *POU2F3*, YAP1 protein has been shown to be heterogeneously expressed across all subtypes [4, 30]. T-cell inflamed signature scores in subtypes defined per the method of Rudin et al. [6] are shown in Fig. S4B.

We evaluated OS with D ± T + EP in patients with each of the SCLC molecular types. In subtypes per the method of Gay et al. [5], we found that SCLC-I showed the greatest median OS benefit with D ± T + EP, at 24.0 months, compared to 12.1 months and 11.5 months with SCLC-N and SCLC-A subtypes, respectively; the SCLC-P subtype defined by expression of *POU2F3* showed the poorest outcome with immunochemotherapy, consistent with previous findings [5], with a median OS of 7.0 months (Fig. 3D). Given sample size limitations, including fewer patients in the EP cohort, there was limited ability to detect differences in OS within subtypes in the D ± T + EP versus EP treatment cohorts. The differential outcome between subtypes was not statistically significant. Nonetheless, the relatively high median OS in patients with SCLC-I compared with other subtypes who received immunochemotherapy is consistent with previous findings [5]. Furthermore, while comparison of OS with D ± T + EP versus EP indicated a trend toward all subtypes except SCLC-P gaining benefit from D ± T + EP (Fig. 3D, Table S5), in the SCLC-I group, 10 patients received D ± T + EP while only two patients received EP, limiting the feasibility of comparing between treatment arms. Numerical differences in OS with D ± T + EP and EP alone in subtypes defined per the method of Rudin et al. [6], are shown in Fig. S4C. In summary, while the trend suggesting greater benefit for the SCLC-I subgroup in the D ± T + EP arms merits further investigation, neither subtyping method was powered to identify patients with the most durable benefit from D + EP or D + T + EP.

**Fig. 4:**
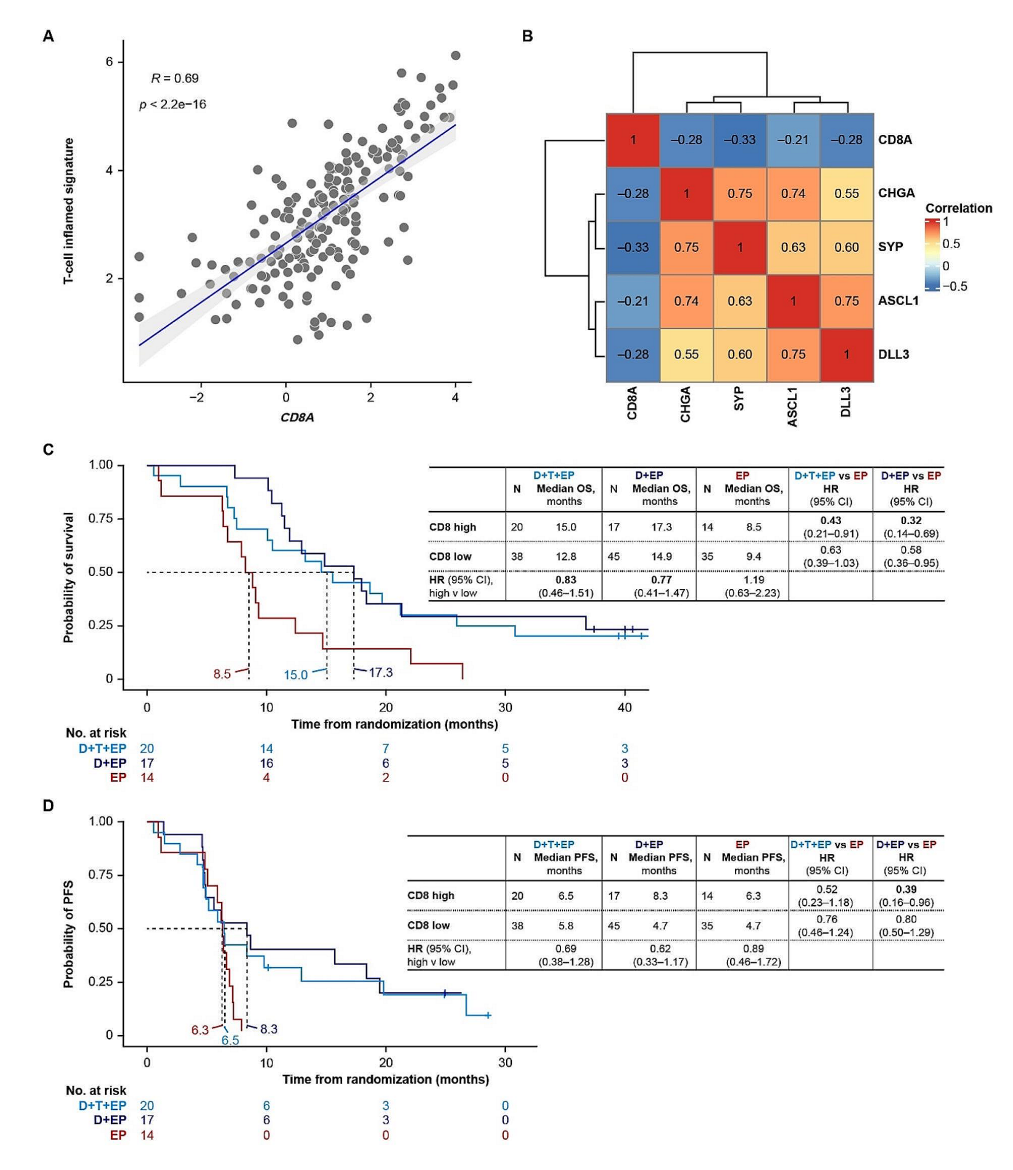
CD8 expression and association with OS in patients receiving D ± T + EP in CASPIAN. (**A**) Correlation of T-cell inflamed signature score with *CD8A* expression (Pearson correlation methodology). (**B**) Inverse correlation of *CD8A* expression and expression of *DLL3* and other neuroendocrine markers (Pearson correlation methodology). (C, D) Kaplan‒Meier analyses of (**C**) OS and (**D**) PFS with D + T + EP and D + EP versus EP in patients with high (top 30% cut-off) CD8 cell density on IHC, and OS/PFS comparisons in patients with high versus low CD8 cell density in the CD8 IHC BEP (*n* = 169). BEP, biomarker-evaluable population; CI, confidence interval; D, durvalumab; EP, etoposide-platinum; HR, hazard ratio; IHC, immunohistochemistry; OS, overall survival; PFS, progression-free survival; T, tremelimumab

We therefore looked for alternative gene expression biomarkers associated with improved outcomes with D ± T + EP. Gene expression profiles of patients in the RNAseq BEP according to OS of ≥ 18 or < 18 months are shown in Fig. S5. Consistent with the greatest benefit with D ± T + EP being seen in the SCLC-I subtype, analysis of OS according to high versus low T-cell inflamed signature score demonstrated greater magnitudes of OS difference with D + T + EP in particular (median OS 30.8 vs. 10.0 months, high vs. low score; HR = 0.36; 95% CI, 0.18–0.72) and with D + EP (median 15.8 vs. 11.5 months; HR = 0.64; 95% CI, 0.31–1.31) than with EP (median 9.1 vs. 8.3 months; HR = 0.93; 95% CI, 0.52–1.66) (Fig. S6A). Of the 18 genes in the T-cell inflamed signature [22, 23], we found that *CD8A* expression was highly correlated with the overall T-cell inflamed signature score (Fig. 4A) and inversely associated with neuroendocrine markers and *DLL3* expression (Fig. 4B), and so we evaluated outcomes specifically according to expression of this marker. Reflecting findings by overall T-cell inflamed signature score, patients in the top quartile (versus the rest) of *CD8A* expression had prolonged OS with D + T + EP (median 25.1 vs. 10.0 months; HR = 0.50; 95% CI, 0.27–0.93) and numerically longer OS with D + EP (median 16.3 vs. 10.6 months; HR = 0.58; 95% CI, 0.29–1.17) but similar OS with EP (median 9.1 vs. 8.3 months; HR = 0.84; 95% CI, 0.45–1.59) (Fig. S6B).

We validated this association in gene expression at the protein level by conducting IHC analysis for CD8 in pretreatment tumor biospecimens. Analysis of CD8 cell density distribution by IHC in the CD8 IHC BEP showed that intermediate (100–500 cells/mm^2^) and high (> 500 cells/mm^2^) densities were present in 35.6% and 10.9% of patients, respectively, with the remaining 53.4% having densities < 100 cells/mm^2^. The importance of CD8 T cells in response to immunotherapy with durvalumab is illustrated by analysis of OS and PFS in the CD8 IHC BEP subgroup (Fig. 4C); these data showed that patients with high CD8 cell density by IHC (top 30%) had prolonged OS with D + T + EP versus EP (median 15.0 vs. 8.5 months; HR = 0.43; 95% CI, 0.21–0.91) and with D + EP versus EP (median 17.3 vs. 8.5 months; HR = 0.32; 95% CI, 0.14–0.69), but that there was no further OS benefit from the addition of tremelimumab to D + EP in this population (median 15.0 vs. 17.3 months, D + T + EP vs. D + EP) (Fig. 4C). Furthermore, OS was numerically longer in patients with high versus low CD8 cell density on IHC in both the D + T + EP (median 15.0 vs. 12.8 months; HR = 0.83; 95% CI, 0.46–1.51) and D + EP (median 17.3 vs. 14.9 months; HR = 0.77; 95% CI, 0.41–1.47) groups but not with EP (median 8.5 vs. 9.4 months; HR = 1.19; 95% CI, 0.63–2.23) (Fig. 4C). Similar trends were observed for PFS (Fig. 4D).

### Gene expression profiling reveals *CD4* and APM signature expression associated with OS benefit from addition of anti-CTLA-4 immunotherapy to D + EP

Given the absence of a difference in OS with D + T + EP and D + EP in the CD8-high subset, we next analyzed gene expression in patients sensitive specifically to D + T + EP with the aim of identifying biomarkers of benefit from the addition of tremelimumab to D + EP. A total of 295 highly expressed genes were identified.

Amongst these highly expressed genes, *CD4* – which is associated with CTLA-4 biology – was present and was shown to associate with increased OS benefit in the D + T + EP arm in the RNAseq BEP; median OS was 25.9 versus 10.4 months (HR = 0.48; 95% CI, 0.23–0.99) in patients with high versus low expression of *CD4* (Fig. S6C). In addition, similar trends were seen for *CTLA-4* itself (Fig. S6D) and *FOXP3* (Fig. S6E); in the D + T + EP arm, median OS was 22.8 versus 10.4 months (HR = 0.58; 95% CI, 0.31–1.07) in patients with high versus low expression of *CTLA-4*, and 28.4 versus 10.4 months (HR = 0.57; 95% CI, 0.29–1.14) in patients with high versus low expression of *FOXP3*. In contrast, expression of these genes did not impact OS in the D + EP arm, confirming a specific role in the mechanisms of CTLA-4 blockade.

**Fig. 5:**
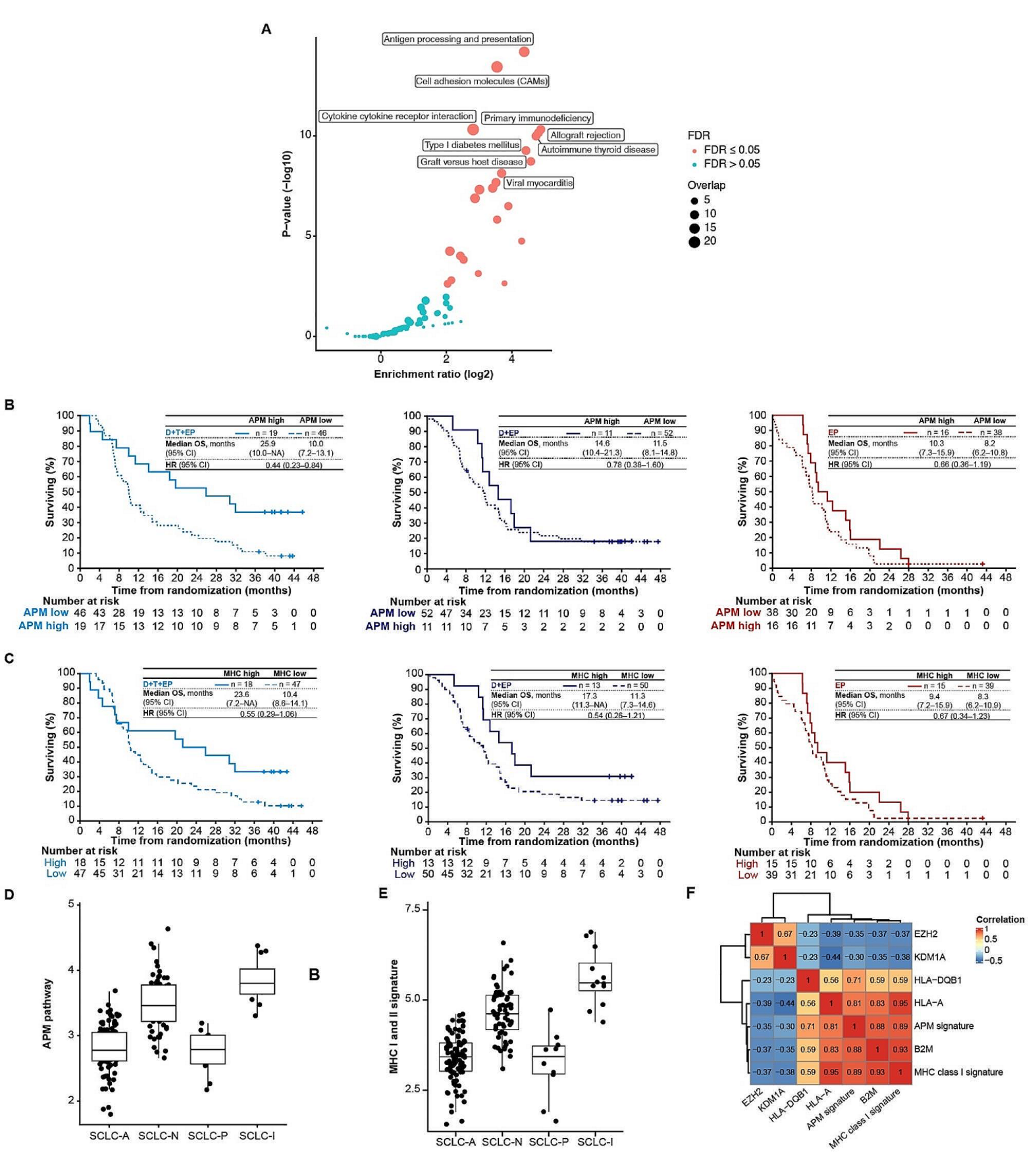
APM gene signature and its surrogate, MHC I and II, associate with OS with D + T + EP. (**A**) Gene expression signatures in the MSigDB enriched in patients benefitting from D + T + EP, including the APM signature (KEGG gene set). (**B**, **C**) OS by treatment arm and expression of (**B**) APM signature (high [top 25% cut-off] vs. low) and (**C**) MHC I and II signature (high vs. low) in the RNAseq BEP (*n* = 182). The MHC I and II signature included *B2M*, *HLA-A*, *HLA-B*, *HLA-C*, *HLA-DPA1*, *HLA-DPB1*, *HLA-DMA*, *HLA-DMB*, *HLA-DQA1*, *HLA-DQB1*, *HLA-DOA*, *HLA-DOB*, *HLA-DRA*, *HLA-DRB1*, *HLA-DRB5*, *HLA-DQA2*, *HLA-DQB2*, *HLA-E*, *HLA-F*, and *HLA-G*. (**D**, **E**) Expression of (**D**) APM signature and I MHC I and II signature according to SCLC molecular subtype per the method of Gay et al. [5]. (**F**) Inverse correlation of APM gene expression signature with expression of *EZH2* and *LSD1*/*KDM1A* (Pearson correlation methodology)

Signatures from the KEGG pathway derived from MSigDB enriched within the 295 genes are illustrated in Fig. 5A, including the gene set for APM. GSEA of the APM signature by treatment arm is shown in Fig. S7. Evaluation of OS according to the expression of these signatures by treatment arm showed durable OS benefit with D + T + EP in those with high (top 25% cut-off) expression of the APM (median 25.9 months [95% CI, 10.0–not assessable] vs. 10.0 months [95% CI, 7.2–13.1] in those with low expression; Fig. 5B) and MHC I and II (median 23.6 months, vs. 10.4 months in those with low expression; Fig. 5C) signatures. Notably, in the context of previous data by SCLC subtype, we found that APM signature gene expression was higher in the SCLC-I subtype [5] (Fig. 5D); APM signature gene expression was also higher in the SCLC-Y (YAP1) subtype per the method of Rudin et al. [6] (Fig. S8A). Reflecting these data, we also found higher gene expression of the MHC I and II signature in the SCLC-I subtype (Fig. 5E) and the subtype defined by highest relative gene expression of *YAP1* (Fig. S8B). Conversely, expression of *EZH2* and *LSD1/KDM1A*, which mediate transcriptional silencing of MHC I antigen processing [31–33], was inversely correlated with expression of genes in the APM signature (Fig. 5F).

**Fig. 6:**
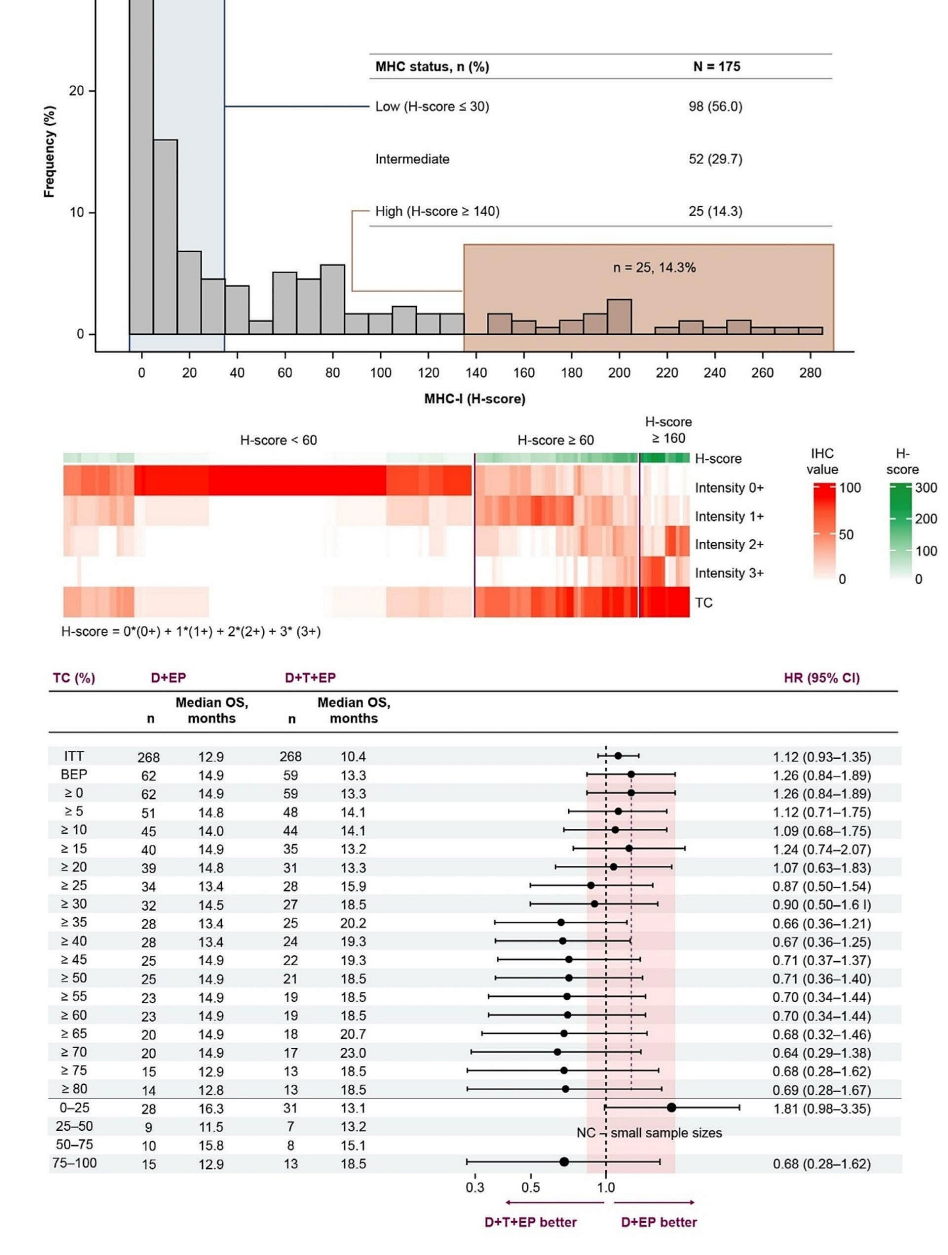
Distribution of MHC I expression by IHC and association of MHC I expression with OS. (**A**) Distribution of MHC I H-score by IHC. (**B**) TC positivity and intensity according to H score. (**C**) OS HRs and 95% CI with D + T + EP versus D + EP according to MHC I expression (%TC) by IHC, MHC I IHC BEP (*n* = 175)

We sought to validate these findings from RNAseq analyses using IHC analysis for MHC I as a surrogate of the APM RNA signature. MHC I H-score distribution and its breakdown to intensity and TC positivity are shown in Fig. 6A, B. Evaluation of OS with D + T + EP versus D + EP according to IHC analysis of MHC I expression showed a trend towards increasing OS benefit with D + T + EP with higher MHC I %TC (Fig. 6C), with HRs for the comparisons of OS between D + T + EP and D + EP ranging from 1.81 (95% CI, 0.98–3.35), favoring D + EP, in patients with MHC I TC of 0–25% to 0.68 (95% CI, 0.28–1.62), favoring D + T + EP, in those with MHC I TC of 75–100%.

## Discussion

Molecular biomarker analyses in nearly 200 patients with ES-SCLC enrolled in the randomized phase 3 CASPIAN trial reveal a mutational landscape consistent with prior analyses of SCLC [2, 3]. As expected, we found both *TP53* and *RB1* to be mutated in most tumors. RB1 loss-of-function prevalence reported here may be an underestimate based on prior detailed analyses of *RB1* status in SCLC, which suggested that the actual rate of RB1 inactivation in SCLC is approximately 94% [34]. However, the intertumoral mutational landscape of SCLC is otherwise heterogeneous, with the frequency of other gene mutations dropping dramatically compared with *TP53* and *RB1*. Compilation of multiple large datasets such as ours are valuable for exploring and categorizing this heterogeneity, and for assessing drivers of response and/or resistance to immunotherapy. Mutations in *TP53* have previously been associated with improved outcome following immunotherapy-containing regimens in NSCLC [35], and prior analyses of a possible association with *RB1* mutational status and immunotherapy outcomes have yielded conflicting evidence, which might be in part attributable to methodologic differences in assessing RB1 functional status [34, 36]. In the context of the analysis presented here, mutations in neither gene impacted the outcome in either of the immunotherapy-containing arms in CASPIAN.

As we report herein, the mean tTMB in patients with ES-SCLC in CASPIAN was comparable to that in patients with metastatic NSCLC in the phase 3 MYSTIC [28] and NEPTUNE [29] studies. However, per the previous analysis of CASPIAN [16], and in contrast to findings in advanced NSCLC [13], evaluation of outcomes in patient subgroups defined according to a tTMB cut-off of 10 mut/Mb did not show a difference in OS. Exploratory correlative analysis of the Impower133 study according to TMB in blood (bTMB) also did not show significant differences in outcomes using cut-offs of 10 or 16 mut/Mb, although the HR for OS favored the atezolizumab arm in patients with bTMB ≥ 16 mut/Mb (0.58) more strongly than in patients with bTMB < 16 mut/Mb (0.79) [17]. We can therefore conclude only that the high mutational burden in ES-SCLC [37] is similar to that in mNSCLC but appears to have less of an impact on outcomes with immunotherapy.

The immune phenotype and contexture is important for response to immunotherapy [37], with different tumor types comprising differing proportions of inflamed, immune-excluded, and immune-desert phenotypes. In ES-SCLC, we and others have shown that PD-L1 TC expression levels are markedly low [17], relative to the expression seen in NSCLC, in which it serves as an informative biomarker [13, 14, 28]. Of note, our data from CASPIAN indicate that PD-L1 IC expression ≥ 1% is more prevalent in ES-SCLC (25.8%) compared to PD-L1 TC ≥ 1% (5.7%), and thus PD-L1 on immune cells or immune cells themselves could be more important in driving clinical benefit with D ± T + EP in the subset of patients with PD-L1 TC/IC ≥ 1% in CASPIAN. These differences support that the biology of the tumor type is of relevance in determining response to immunotherapy treatment, and poses the question of whether we can identify the contribution of the immune contexture beyond PD-L1 in response to immunotherapy.

Our analyses of OS in CASPIAN by SCLC molecular subtypes in the RNAseq BEP are underpowered but show trends supporting the prior findings from the analysis of Impower133 by Gay et al. [5] and demonstrating that the highest OS benefit from immunochemotherapy with D ± T + EP was seen in the SCLC-I subtype associated with an inflamed gene signature. Our data with both D ± T + EP and EP alone also validate the observation from the analysis of Impower133 of a particularly poor outcome in the SCLC-P subtype [5], supporting the suggestion that this may be generally a marker of poor prognosis. Furthermore, we showed that, in subtypes defined by highest relative gene expression of the single transcription factors *ASCL1, NEUROD1, POU2F3*, and *YAP1*, per Rudin et al. [6], greatest numerical OS benefit with D + EP was observed in the SCLC-Y (YAP1) subtype, which is also associated with an inflamed phenotype and other markers of clinical benefit with immunotherapy [21, 22]. While these subtype classifications largely overlap with each other [21], the SCLC-Y subtype has been called into question due to YAP1 protein expression not distinguishing a distinct subset of tumors [1, 4, 5, 24], which may result in divergent subtype classifications according to methodology. We therefore primarily focused on the former subtype classification in this paper [4–6].

Although the greatest median OS benefit from immunochemotherapy with D ± T + EP was seen in the SCLC-I and SCLC-Y (YAP1) subtypes, numerical benefit from immunochemotherapy versus EP was seen in other subtypes, except SCLC-P, consistent with prior findings. Therefore, we sought to define additional immunologic markers that might be more specifically associated with benefit from D + T + EP and D + EP in patients with SCLC. Consistent with other findings [5], we showed that the SCLC-I subtype had an elevated T-cell inflamed signature and an elevated APM signature, which are associated with response to immunotherapy [5, 23]. It is well known that CD8 cytotoxic T cells play a key role in the antitumor effect observed with immune checkpoint blockade [38], with inhibition of the PD-1/PD-L1 axis allowing for prolonged antitumor CD8 activity. Thus, in this context, we found that there was a greater PFS and OS benefit with both D + EP and D + T + EP versus EP alone in patients with high CD8 cell density compared with in the remaining (CD8-low) population, suggesting CD8 as a marker that may be more specifically aligned with benefit from blockade of the PD-1/PD-L1 axis in ES-SCLC. As expected in the context of the mechanisms of action of durvalumab and tremelimumab, CD8 expression did not differentiate between the outcomes achieved among patients receiving D + T + EP or D + EP; thus, our data support the hypothesized mechanism of action of durvalumab and PD-L1 inhibition more broadly and suggest that CD8 may be a biomarker for benefit with these agents.

We also aimed to identify biomarkers specific for additional OS benefit from CTLA-4 blockade with tremelimumab with the investigation of additional potential drivers of benefit from dual immunotherapy. We analyzed genes highly expressed in patients who were sensitive to D + T + EP and found that APM gene expression was enriched in these patients. It is well known that the APM is crucial for the antitumor activity of the immune system, with loss or downregulation of the APM being an immune escape mechanism for tumor growth [39] and genetic, transcriptomic, or epigenetic disruption of APM seen commonly across cancer types [40].

In findings that demonstrate the hypothesized mechanism of action of tremelimumab, we showed that high MHC I and II gene expression measured by RNAseq and MHC I %TC expression via IHC were associated with specific survival benefit with D + T + EP (versus D + EP). These findings are supported by previous analyses in SCLC cell lines and responders to immunotherapy that identified MHC I as a biomarker of SCLC immune responsiveness and durable benefit from immune checkpoint blockade [20, 25]. Although the mechanism is not fully understood, CTLA-4 inhibition may, as noted earlier, lower the priming threshold for new T cell activation and has been shown to increase the diversity of the T-cell receptor repertoire [41], potentially enabling a response to a greater range of tumour neoantigens. In the context of treatment with chemotherapy (EP) and anti-PD-L1 therapy (D), the mechanism of CTLA-4 blockade may be able to uniquely harness enhanced antigen presentation in a way that anti-PD-L1 alone cannot.

In addition to expression of APM genes, high *CD4*, *FOXP3*, and *CTLA-4* expression all conferred long-term benefit from the addition of tremelimumab to D + EP. All three of these genes are suggestive of a key role for CD4 T cells in the response to tremelimumab. This is in keeping with studies showing that one of the key differentiating effects of the CTLA-4 versus the PD-1 pathway is the diversification of CD4 phenotype [42, 43], in particular the expansion of potential effector CD4 cells. Recent studies have highlighted the potential of these CD4 cells to not only support CD8 activation but to mediate direct cytotoxic killing of MHC-II-expressing tumour cells [44]. Intriguingly, preliminary exploratory analysis of germline whole exome sequencing data from CASPIAN identified the presence of a specific allele of MHC II as enriching for tremelimumab benefit [45]. While the mechanism for the particular allele identified, DQB1*03:01, remains unclear, it is possible that specific neoantigens presented in this context may be enabling such cytotoxic CD4 activity.

As only a subset of SCLC patients have long-term responses to current anti-PD-(L)1 immunotherapy a personalized approach to treatment is essential, and our findings are important in the context of this need for potential biomarkers of better or poorer outcomes with immunotherapy. Personalized therapy for SCLC could potentially be tailored based upon such biomarkers [7], utilizing novel approaches beyond current immunotherapy options such as targeting novel immune checkpoints [46] or using bispecific T-cell engaging antibodies [47]. One approach may be to improve immune system activity. Epigenetic silencing of the APM may be the relevant mechanism of immune escape in SCLC, and it has been shown that epigenetic modulation can increase MHC I expression in SCLC [25, 32, 33], whereas expression of the epigenetic regulators *EZH2* and *LSD1* has been shown to be inversely correlated with outcomes with immunotherapy [21]. In this context, cfDNA methylomics may be a valuable technology for understanding kinetics of SCLC heterogeneity and personalizing treatment for patients [48], potentially through the use of EZH2 or LSD1 inhibition [25, 33]. A related approach may be to identify SCLC-specific targets and use distinct agents to target/treat the disease [49]. As shown in our analyses, neuroendocrine markers and *DLL3* are inversely correlated with inflamed SCLC tumors and may indicate the need for a different treatment approach, either independently or in combination with immunotherapy [47]. Bispecific T-cell engaging antibodies targeting DLL3 [50], such as tarlatamab [47], may be particularly beneficial for tailoring therapy for the neuroendocrine SCLC-A and SCLC-N subtypes, in which *DLL3* expression is higher and MHC I expression is lower. A phase 1 study of tarlatamab in combination with durvalumab or atezolizumab plus etoposide-carboplatin in ES-SCLC is currently ongoing (NCT05361395). Finally, our data support the poor clinical outcome in the distinct SCLC-P subtype characterized by *POU2F3* expression, highlighting the need for novel therapeutic targets for this subtype and potentially for routine stratification by subtype.

In conclusion, our findings have revealed, for the first time in a large cohort of patients with ES-SCLC, biomarkers associated with the hypothesized mechanisms of action of durvalumab and tremelimumab that are important for improved outcomes with immunotherapy. Not only have we demonstrated that the tumor microenvironment is an important factor mediating better outcomes with D ± T + EP in ES-SCLC, but also we have identified canonical immune markers defining patient subsets that respond to durvalumab and/or durvalumab plus tremelimumab in combination with chemotherapy. We have shown that long-term benefit from immunotherapy is observed in a subset of patients whose tumor immune microenvironment is primed to benefit; however, these findings also serve to highlight the ongoing need to have personalized medicine approaches in SCLC to further improve the outcome of patients with ES-SCLC.

### Electronic supplementary material

Below is the link to the electronic supplementary material.

Supplementary materials: Supplementary Methods, Supplementary References (8), 4 Supplementary Tables S1‒5, 10 Supplementary Figures S1‒10, Plain Language Summary.

## Data Availability

Further information and requests for resources and data, including RNA sequencing data, should be directed to and will be fulfilled by the lead contact, Yashaswi Shrestha (email: yashaswi.shrestha@astrazeneca.com). Additionally, data underlying the findings described in this manuscript may be obtained in accordance with AstraZeneca’s data sharing policy described at https://astrazenecagrouptrials.pharmacm.com/ST/Submission/Disclosure. Data for studies directly listed on Vivli can be requested through Vivli at www.vivli.org. Data for studies not listed on Vivli could be requested through Vivli at https://vivli.org/members/enquiries-about-studies-not-listed-on-the-vivli-platform/. AstraZeneca’s Vivli member page is also available outlining further details: https://vivli.org/ourmember/astrazeneca/.

## Declarations

### Inclusion and diversity statement

We support inclusive, diverse, and equitable conduct of research. We worked to ensure gender balance in the recruitment of human subjects. We worked to ensure ethnic or other types of diversity in the recruitment of human subjects. We worked to ensure that the study questionnaires were prepared in an inclusive way. One or more of the authors of this paper self-identifies as a member of the LGBTQIA + community. One or more of the authors of this paper self-identifies as a gender minority in their field of research. While citing references scientifically relevant for this work, we also actively worked to promote gender balance in our reference list.

## Abbreviations

APM: Antigen processing and presentation machinery
BEP: Biomarker-evaluable population
bTMB: Blood TMB
Cat: Catalogue number
CI: Confidence interval
CNL: Cognition Network Language
CR: Complete response
CTLA-4: Cytotoxic T-lymphocyte-associated antigen 4
D: Durvalumab
DLL3: Delta-like ligand 3
EDTA: Ethylenediaminetetraacetic acid
EP: Etoposide-platinum
ES-SCLC: Extensive-stage small-cell lung cancer
EXP: Expression
FDR: False discovery rate
FFPE: Formalin-fixed paraffin-embedded
FMI: Foundation Medicine, Inc., group analyzed using FoundationOne^®^ CDx assay
GSEA: Gene set enrichment analysis
HLA: Human leukocyte antigen
HR: Hazard ratio
HRP: Horseradish peroxidase
IC: Immune cells
ICI: Immune checkpoint inhibitor
IHC: Immunohistochemistry
IO: Immunotherapy
ITT: Intent-to-treat
KEGG: Kyoto Encyclopedia of Genes and Genomes
MHC: Major histocompatibility complex
mut: Mutated/mutations
mut/Mb: Mutations per megabase
MsigDB: Molecular Signature Database
N/A: Not applicable
NA: Not available
NC: Not calculated
NES: Normalized enrichment score
NGS: Next-generation sequencing
NSCLC: Non-small-cell lung cancer
OS: Overall survival
PCI: Prophylactic cranial irradiation
PD: Progressive disease
PD-L1: Programmed cell death ligand-1
PFS: Progression-free survival
PR: Partial response
PS: Performance status
Q3/4W: Every 3/4 weeks
RECIST: Response Evaluation Criteria in Solid Tumors
RIN: RNA Integrity Number
RNAseq: RNA sequencing
SCLC: Small-cell lung cancer
SD: Stable disease
sig: Signature
T: Tremelimumab
TCR: T-cell receptor
TC: Tumor cells
TMB: Tumor mutational burden
TME: Tumor microenvironment
tTMB: Tissue TMB
WHO: World Health Organization
wt: Wild-type
WTS: Whole-transcriptome sequencing
