# Supplementary material for: Molecular classification and biomarkers of outcome with immunotherapy in extensive-stage small-cell lung cancer: analyses of the CASPIAN phase 3 study
Source: Mol Cancer. 2024 May 30;23:115. doi: 10.1186/s12943-024-02014-x (PMC11137956; doi:10.1186/s12943-024-02014-x)
Supplement: Supplementary file 1 — Supplementary materials: Supplementary Methods, Supplementary References (8), 4 Supplementary Tables S1‒5, 10 Supplementary Figures S1‒10, Plain Language Summary. [file 12943_2024_2014_MOESM1_ESM.docx]

**Molecular classification and biomarkers of outcome with immunotherapy in extensive-stage small-cell lung cancer: analyses of the CASPIAN phase 3 study**

**Additional File 1. Supplementary Methods, Tables, and Figures, and Plain Language Summary**

**Supplementary Methods**

**RNA extraction and whole-transcriptome sequencing (WTS) library preparation**

Sectioned formalin-fixed paraffin-embedded (FFPE) tumor samples were used for total RNA extraction. Total RNA was extracted using the OMEGA M2551 Mag Bind FFPE RNA 96 kit via the KingFisher Flex system and eluted in 50 mL of nuclease-free water. RNA concentration, RNA Integrity Number (RIN), and %DV200 (percentage of RNA fragments >200 nucleotides in size) were determined using Agilent RNA Screentapes or Agilent High Sensitivity RNA Screentapes via Agilent TapeStation. Total RNA was arrayed on a 96-well polymerase chain reaction plate, and WTS libraries were generated using the KAPA RNA HyperPrep Kit with RiboErase (HMR) Globin (Roche cat# KK8563) per the manufacturer’s protocol. Library concentrations were determined using Agilent D1000 screentapes on Agilent TapeStation. WTS libraries were sequenced with the Illumina NovaSeq 6000 kit v1.5 (300 cycles; 150 x 2), and 150 bp paired-end reads were generated, with ~200M reads per sample.

**RNAseq process**

Reads were aligned to the UCSC GRCh38 Homo Sapiens genome build, augmented with transcript information from Ensembl release 86 using STAR (v2.6.1) [1]. Alignments were evaluated for evenness of coverage, ribosomal RNA content, genomic context of alignments, and complexity using a combination of FastQC and Qualimap [2]. Transcripts Per Million measurements per isoform were quantified from the alignments using Salmon (version 1.4) [3] and used to estimate abundance of genes.

**SCLC subtyping**

SCLC subtyping was defined per the method of Gay et al [4] based on differential expression of three transcription factor genes (*ASCL1*, *NEUROD1*, *POU2F3* – respectively, SCLC-A, SCLC-N, and SCLC-P subtypes) and, in those with low/no expression of these genes, an immunologically ‘inflamed’ gene expression pattern (SCLC-I subtype). Subtyping was also defined per the method of Rudin et al [5] based on highest relative expression of single transcription factors (*ASCL1, NEUROD1, POU2F3, YAP1*) – SCLC-A, SCLC-N, SCLC-P, and SCLC-Y subtypes. Per this classification method, the highest relative expression among the four transcription factors determined the subtype, with *ASCL1* and *NEUROD1* subtypes classified as neuroendocrine and *POU2F3* and *YAP1* subtypes lacking neuroendocrine markers. As *YAP1* tends to be expressed in all subtypes, albeit at a relatively low level, a sample is classified as *YAP1* (SCLC-Y subtype) when the relative expression of *YAP1* is higher than that of *ASCL1*, *NEUROD1*, and *POU2F3*.

**T-cell inflamed gene expression signature**

The 18-gene T-cell inflamed gene expression signature, which contains interferon-γ-responsive genes related to antigen presentation, chemokine expression, cytotoxic activity, and adaptive immune resistance, was evaluated and the T-cell inflamed signature score calculated for each sample as previously described [6, 7]. Briefly, the score is determined based on the weighted sum of normalized expression values of *PSMB10*, *HLA-DQA1*, *HLA-DRB1*, *CMKLR1*, *HLA-E*, *NKG7*, *CD8A*, *CCL5*, *CXCL9*, *CD27*, *CXCR6*, *IDO1*, *STAT1*, *TIGIT*, *LAG3*, *CD274*, *PDCD1LG2*, and *CD276*.

**CD8 immunohistochemistry image analysis**

Receiving inspection was done for all slides individually using proprietary software. The slides were visually assessed for scanning, staining and preparation artifacts. Digitally scanned whole-slide images were analyzed using an image analysis pipeline executed in Python, version 3.8, and Definiens Developer XD 2.8 (Definiens, Munich, Germany). Deep-learning algorithms were implemented in TensorFlow. Invasive tumor regions of interest and major artefact exclusions were manually annotated by a pathologist in the proprietary software. Using a Deep Learning-based semantic segmentation algorithm – an Unet [8] with ResNet50 encoder [9] – posteriors with classification probabilities of pixels for membranes, cytoplasm and nuclei were generated. Centers of gravity of individual cells were identified applying nonmaximum suppression on the posterior for cell centers. Training of the Deep Learning-based algorithm was performed as described in Kinneer et al [10]. Based on the posteriors derived from the preceding step, diaminobenzidine-stained lymphocytes were segmented and classified using a custom-built rule set implemented in Cognition Network Language (CNL) within the Definiens Developer XD 2.8 framework [11]. Object classification and segmentation in a hierarchical network of cells and regions [12] were saved in a custom .hdf5 file format for subsequent data analysis. Algorithm performance was assessed visually by a pathology informatics specialist. Image analysis results of all processed slides were subject to visual assessment by a Pathology Informatics specialist, where at least 75% of CD8 target cells had to be correctly classified. Samples on which analysis failed were not used for subsequent data analysis.

**REFERENCES**

1. Dobin A, Davis CA, Schlesinger F, Drenkow J, Zaleski C, Jha S, et al. STAR: ultrafast universal RNA-seq aligner. Bioinformatics. 2013;29:15–21.

2. Garcia-Alcalde F, Okonechnikov K, Carbonell J, Cruz LM, Gotz S, Tarazona S, et al. Qualimap: evaluating next-generation sequencing alignment data. Bioinformatics. 2012;28:2678–2679.

3. Patro R, Duggal G, Love MI, Irizarry RA, Kingsford C. Salmon provides fast and bias-aware quantification of transcript expression. Nat Methods. 2017;14:417–419.

4. Gay CM, Stewart CA, Park EM, Diao L, Groves SM, Heeke S, et al. Patterns of transcription factor programs and immune pathway activation define four major subtypes of SCLC with distinct therapeutic vulnerabilities. Cancer Cell. 2021;39:346–360.e347.

5. Rudin CM, Poirier JT, Byers LA, Dive C, Dowlati A, George J, et al. Molecular subtypes of small cell lung cancer: a synthesis of human and mouse model data. Nat Rev Cancer. 2019;19:289–297.

6. Ayers M, Lunceford J, Nebozhyn M, Murphy E, Loboda A, Kaufman DR, et al. IFN-gamma-related mRNA profile predicts clinical response to PD-1 blockade. J Clin Invest. 2017;127:2930–2940.

7. Owonikoko TK, Dwivedi B, Chen Z, Zhang C, Barwick B, Ernani V, et al. YAP1 expression in SCLC defines a distinct subtype with T-cell-inflamed phenotype. J Thorac Oncol. 2021;16:464–476.

8. Ronneberger O, Fischer P, Brox T. U-Net: convolutional networks for biomedical image segmentation. In: Medical Image Computing and Computer-Assisted Intervention – MICCAI 2015. Lecture Notes in Computer Science. Spinger, Cham; 2015. p. 234–241.

9. He K, Zhang X, Ren S, Sun J. Deep residual learning for image recognition. arXiv. 2015:1512.03385v03381.

10. Kinneer K, Wortmann P, Cooper ZA, Dickinson NJ, Masterson L, Cailleau T, et al. Design and preclinical evaluation of a novel B7-H4-directed antibody-drug conjugate, AZD8205, alone and in combination with the PARP1-selective inhibitor AZD5305. Clin Cancer Res. 2023;29:1086–1101.

11. Baatz M, Zimmermann J, Blackmore CG. Automated analysis and detailed quantification of biomedical images using Definiens Cognition Network Technology. Comb Chem High Throughput Screen. 2009;12:908–916.

12. Harder N, Athelogou M, Hessel H, Brieu N, Yigitsoy M, Zimmermann J, et al. Tissue phenomics for prognostic biomarker discovery in low- and intermediate-risk prostate cancer. Sci Rep. 2018;8:4470.

13. Paz-Ares L, Chen Y, Reinmuth N, Hotta K, Trukhin D, Statsenko G, et al. Durvalumab, with or without tremelimumab, plus platinum-etoposide in first-line treatment of extensive-stage small-cell lung cancer: 3-year overall survival update from CASPIAN. ESMO Open. 2022;7:100408.

| **Table S1** Key patient demographics and disease characteristics in the CASPIAN ITT population and in the BEPs for RNA sequencing analysis (RNAseq BEP) and immunohistochemistry analysis of CD8 (CD8 BEP) and MHC I (MHC I BEP) | | | | |
| --- | --- | --- | --- | --- |
| **Characteristic** | **ITT (N=805)** | **RNAseq BEP (n=182)** | **CD8 BEP (n=169)** | **MHC I BEP (n=175)** |
| **Age at study entry** | |  |  |  |
| Median | 63 | 63 | 62 | 62 |
| Range | (28–88) | (36–83) | (36–83) | (36–83) |
| **Sex** |  |  |  |  |
| Male | 576 (71.6%) | 137 (75.3%) | 128 (75.7%) | 128 (73.1%) |
| Female | 229 (28.4%) | 45 (24.7%) | 41 (24.3%) | 47 (26.9%) |
| **WHO PS** |  |  |  |  |
| 0 | 298 (37.0%) | 53 (29.1%) | 55 (32.5%) | 57 (32.6%) |
| 1 | 507 (63.0%) | 129 (70.9%) | 114 (67.5%) | 118 (67.4%) |
| **Metastases at baseline** | |  |  |  |
| Brain | 93 (11.6%) | 18 (9.9%) | 12 (7.1%) | 12 (6.9%) |
| Liver | 329 (40.9%) | 71 (39.0%) | 58 (34.3%) | 64 (36.6%) |
| Pancreatic | 34 (4.2%) | 10 (5.5%) | 11 (6.5%) | 11 (6.3%) |
| **Treatment arm** |  |  |  |  |
| D+T+EP | 268 (33.3%) | 65 (35.7%) | 58 (34.3%) | 59 (33.7%) |
| D+EP | 268 (33.3%) | 63 (34.6%) | 62 (36.7%) | 62 (35.4%) |
| EP | 269 (33.4%) | 54 (29.7%) | 49 (29.0%) | 54 (30.9%) |

| **Table S2** Baseline characteristics and treatment exposure in the ITT population and RNAseq BEP by treatment arm | | | | | | |
| --- | --- | --- | --- | --- | --- | --- |
| **Characteristic** | **ITT** | | | **RNAseq BEP** | | |
|  | **D+T+EP (n=268)** | **D+EP (n=268)** | **EP (n=269)** | **D+T+EP (n=65)** | **D+EP (n=63)** | **EP (n=54)** |
| **Age at study entry** | |  |  |  |  |  |
| Median | 63 | 62 | 63 | 63 | 61 | 63 |
| Range | (36–88) | (28–82) | (35–82) | (36–83) | (40–81) | (38–78) |
| **Sex** |  |  |  |  |  |  |
| Male | 202 (75.4%) | 190 (70.9%) | 184 (68.4%) | 50 (76.9%) | 50 (79.4%) | 37 (68.5%) |
| Female | 66 (24.6%) | 78 (29.1%) | 85 (31.6%) | 15 (23.1%) | 13 (20.6%) | 17 (31.5%) |
| **WHO PS** | | |  |  |  |  |
| 0 | 109 (40.7%) | 99 (36.9%) | 90 (33.5%) | 20 (30.8%) | 20 (31.7%) | 13 (24.1%) |
| 1 | 159 (59.3%) | 169 (63.1%) | 179 (66.5%) | 45 (69.2%) | 43 (68.3%) | 41 (75.9%) |
| **Metastases at baseline** | |  |  |  |  |  |
| Brain | 38 (14.2%) | 28 (10.4%) | 27 (10.0%) | 11 (16.9%) | 3 (4.8%) | 4 (7.4%) |
| Liver | 117 (43.7%) | 108 (40.3%) | 104 (38.7%) | 27 (41.5%) | 24 (38.1%) | 20 (37.0%) |
| Pancreas | 13 (4.9%) | 11 (4.1%) | 10 (3.7%) | 8 (12.3%) | 1 (1.6%) | 1 (1.9%) |
| **Treatment exposure** | (n=266) | (n=265) | (n=266) | (n=65) | (n=63) | (n=54) |
| Received 5 doses of T | 161 (60.5%) | n/a | n/a | 49 (75.4%) | n/a | n/a |
| Median doses of D | 6 | 7 | n/a | 7 | 7 | n/a |
| Carboplatin/cisplatin* | 202/66 (75.9/24.8%) | 208/65 (78.0/24.5%) | 208/67 (78.0/25.0%) | 51/17 (78.5/26.2%) | 53/13 (84.1/20.6%) | 44/16 (81.5/29.6%) |
| Received ≥4 EP cycles^†^ | 215/264 (81.4%) | 230 (86.8%) | 225 (84.6%) | 61 (93.8%) | 56 (88.9%) | 46 (85.2%) |

*Patients were allowed to switch between carboplatin and cisplatin at the investigator’s discretion. ^†^Based on etoposide exposure.

| **Table S3** Baseline characteristics and treatment exposure in the ITT population and CD8 IHC BEP by treatment arm | | | | | | |
| --- | --- | --- | --- | --- | --- | --- |
| **Characteristic** | **ITT** | | | **CD8 BEP** | | |
|  | **D+T+EP (n=268)** | **D+EP (n=268)** | **EP (n=269)** | **D+T+EP (n=58)** | **D+EP (n=62)** | **EP (n=49)** |
| **Age at study entry** | |  |  |  |  |  |
| Median | 63 | 62 | 63 | 63 | 61 | 63 |
| Range | (36–88) | (28–82) | (35–82) | (36–83) | (40–81) | (38–78) |
| **Sex** |  |  |  |  |  |  |
| Male | 202 (75.4%) | 190 (70.9%) | 184 (68.4%) | 45 (77.6%) | 48 (77.4%) | 35 (71.4%) |
| Female | 66 (24.6%) | 78 (29.1%) | 85 (31.6%) | 13 (22.4%) | 14 (22.6%) | 14 (28.6%) |
| **WHO PS** | | |  |  |  |  |
| 0 | 109 (40.7%) | 99 (36.9%) | 90 (33.5%) | 16 (27.6%) | 24 (38.7%) | 15 (30.6%) |
| 1 | 159 (59.3%) | 169 (63.1%) | 179 (66.5%) | 42 (72.4%) | 38 (61.3%) | 34 (69.4%) |
| **Metastases at baseline** | |  |  |  |  |  |
| Brain | 38 (14.2%) | 28 (10.4%) | 27 (10.0%) | 6 (10.3%) | 3 (4.8%) | 3 (6.1%) |
| Liver | 117 (43.7%) | 108 (40.3%) | 104 (38.7%) | 21 (36.2%) | 19 (30.6%) | 18 (36.7%) |
| Pancreas | 13 (4.9%) | 11 (4.1%) | 10 (3.7%) | 8 (13.8%) | 2 (3.2%) | 1 (2.0%) |
| **Treatment exposure** | (n=266) | (n=265) | (n=266) | (n=58) | (n=62) | (n=49) |
| Received 5 doses of T | 161 (60.5%) | n/a | n/a | 45 (77.6%) | n/a | n/a |
| Median doses of D | 6 | 7 | n/a | 7 | 8 | n/a |
| Carboplatin/cisplatin* | 202/66 (75.9/24.8%) | 208/65 (78.0/24.5%) | 208/67  (78.0/25.0%) | 47/14 (81.0/24.1%) | 50/14 (80.6/22.6%) | 39/15 (79.6/30.6%) |
| Received ≥4 EP cycles^†^ | 215/264 (81.4%) | 230 (86.8%) | 225 (84.6%) | 53 (91.4%) | 57 (91.9%) | 42 (85.7%) |

*Patients were allowed to switch between carboplatin and cisplatin at the investigator’s discretion. ^†^Based on etoposide exposure.

| **Table S4** Baseline characteristics and treatment exposure in the ITT population and MHC I IHC BEP by treatment arm | | | | | | |
| --- | --- | --- | --- | --- | --- | --- |
| **Characteristic** | **ITT** | | | **MHC I BEP** | | |
|  | **D+T+EP (n=268)** | **D+EP (n=268)** | **EP (n=269)** | **D+T+EP (n=59)** | **D+EP (n=62)** | **EP (n=54)** |
| **Age at study entry** | |  |  |  |  |  |
| Median | 63 | 62 | 63 | 63 | 61 | 63 |
| Range | (36–88) | (28–82) | (35–82) | (36–83) | (40–81) | (38–79) |
| **Sex** |  |  |  |  |  |  |
| Male | 202 (75.4%) | 190 (70.9%) | 184 (68.4%) | 46 (78.0%) | 46 (74.2%) | 36 (66.7%) |
| Female | 66 (24.6%) | 78 (29.1%) | 85 (31.6%) | 13 (22.0%) | 16 (25.8%) | 18 (33.3%) |
| **WHO PS** | | |  |  |  |  |
| 0 | 109 (40.7%) | 99 (36.9%) | 90 (33.5%) | 16 (27.1) | 24 (38.7%) | 17 (31.5%) |
| 1 | 159 (59.3%) | 169 (63.1%) | 179 (66.5%) | 43 (72.9) | 38 (61.3%) | 37 (68.6%) |
| **Metastases at baseline** | |  |  |  |  |  |
| Brain | 38 (14.2%) | 28 (10.4%) | 27 (10.0%) | 6 (10.2%) | 3 (4.8%) | 3 (5.6%) |
| Liver | 117 (43.7%) | 108 (40.3%) | 104 (38.7%) | 23 (39.0%) | 18 (29.0%) | 23 (42.6%) |
| Pancreas | 13 (4.9%) | 11 (4.1%) | 10 (3.7%) | 8 (13.6%) | 2 (3.2%) | 1 (1.9%) |
| **Treatment exposure** | (n=266) | (n=265) | (n=266) | (n=59) | (n=62) | (n=54) |
| Received 5 doses of T | 161 (60.5%) | n/a | n/a | 45 (76.3%) | n/a | n/a |
| Median doses of D | 6 | 7 | n/a | 7 | 8 | n/a |
| Carboplatin/cisplatin* | 202/66 (75.9/24.8%) | 208/65 (78.0/24.5%) | 208/67 (78.0/25.0%) | 47/15 (79.7/25.4%) | 50/14 (80.6/22.6%) | 42/17 (77.8/31.5%) |
| Received ≥4 EP cycles^†^ | 215/264 (81.4%) | 230 (86.8%) | 225 (84.6%) | 53 (89.8%) | 56 (90.3%) | 47 (87.0%) |

*Patients were allowed to switch between carboplatin and cisplatin at the investigator’s discretion. ^†^Based on etoposide exposure

| **Table S5** OS and PFS by treatment group and individual SCLC molecular subtype per the method of Gay et al [4] (SCLC-A, SCLC-N, SCLC-P, and SCLC-I subtypes). | | | | |
| --- | --- | --- | --- | --- |
| **Treatment group** | **SCLC subtype** | | | |
|  | **SCLC-A** | **SCLC-N** | **SCLC-P** | **SCLC-I** |
| **D+T+EP** | **n = 34** | **n = 21** | **n = 3** | **n = 7** |
| Median OS, months (95% CI) | 12.5 (8.6–15.8) | 11.4 (7.5–25.9) | 7.2 (5.0–NA) | 30.8 (1.9–NA) |
| Median PFS, months (95% CI) | 5.0 (4.6–6.5) | 6.5 (4.6–9.8) | 2.9 (1.6–NA) | 5.9 (1.9–NA) |
| **D+EP** | **n = 37** | **n = 20** | **n = 3** | **n = 3** |
| Median OS, months (95% CI) | 11.3 (7.3–14.8) | 13.0 (8.6–21.3) | 6.8 (2.9–NA) | 17.3 (12.8–NA) |
| Median PFS, months (95% CI) | 4.7 (4.6–5.4) | 4.7 (3.5–6.6) | 2.9 (1.6–NA) | 6.8 (6.2–NA) |
| **EP** | **n = 25** | **n = 23** | **n = 4** | **n = 2** |
| Median OS, months (95% CI) | 8.1 (4.8–11.2) | 10.5 (7.9–12.4) | 6.8 (1.3–NA) | 16.3 (6.3–NA) |
| Median PFS, months (95% CI) | 4.6 (2.9–6.4) | 6.3 (4.7–7.2) | 2.1 (1.3–NA) | 6.3 (NA–NA) |

NA, not assessable.

**Fig. S1** OS in the CASPIAN ITT population and in the BEPs for RNA sequencing and IHC analyses. OS in the (A) ITT population [13], (B) RNAseq BEP, (C) CD8 BEP, and (D) MHC I BEP.

[Adapted from reference 13, published under Creative Commons CC-BY-NC-ND license, © the Authors]


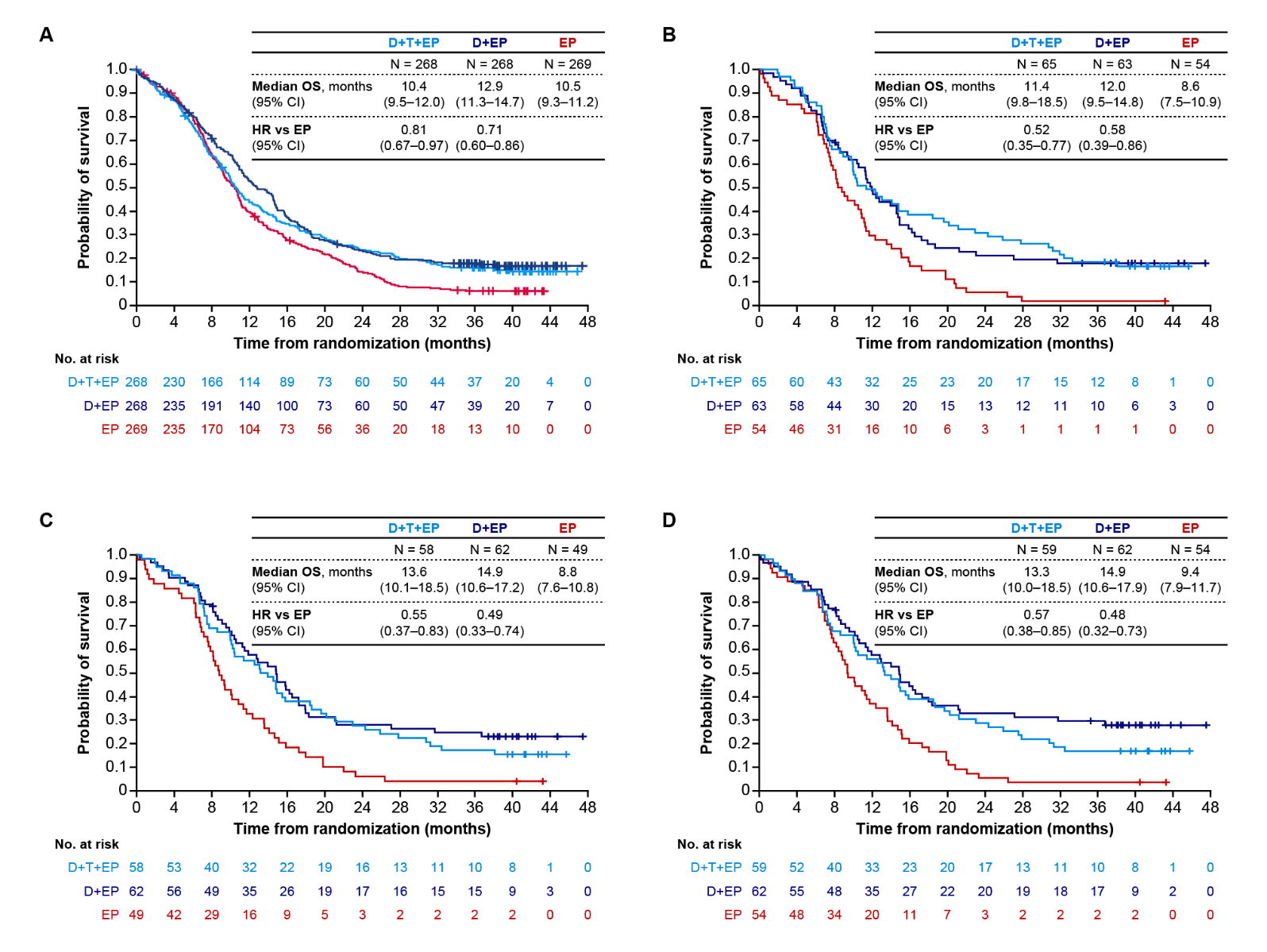


**Fig. S2** Common mutations in the CASPIAN population do not inform outcomes with immunotherapy. Kaplan–Meier analyses of OS with immunotherapy (IO; D±T) plus EP or EP in CASPIAN according to mutational status of (A) *TP53* and (B) *RB1*.


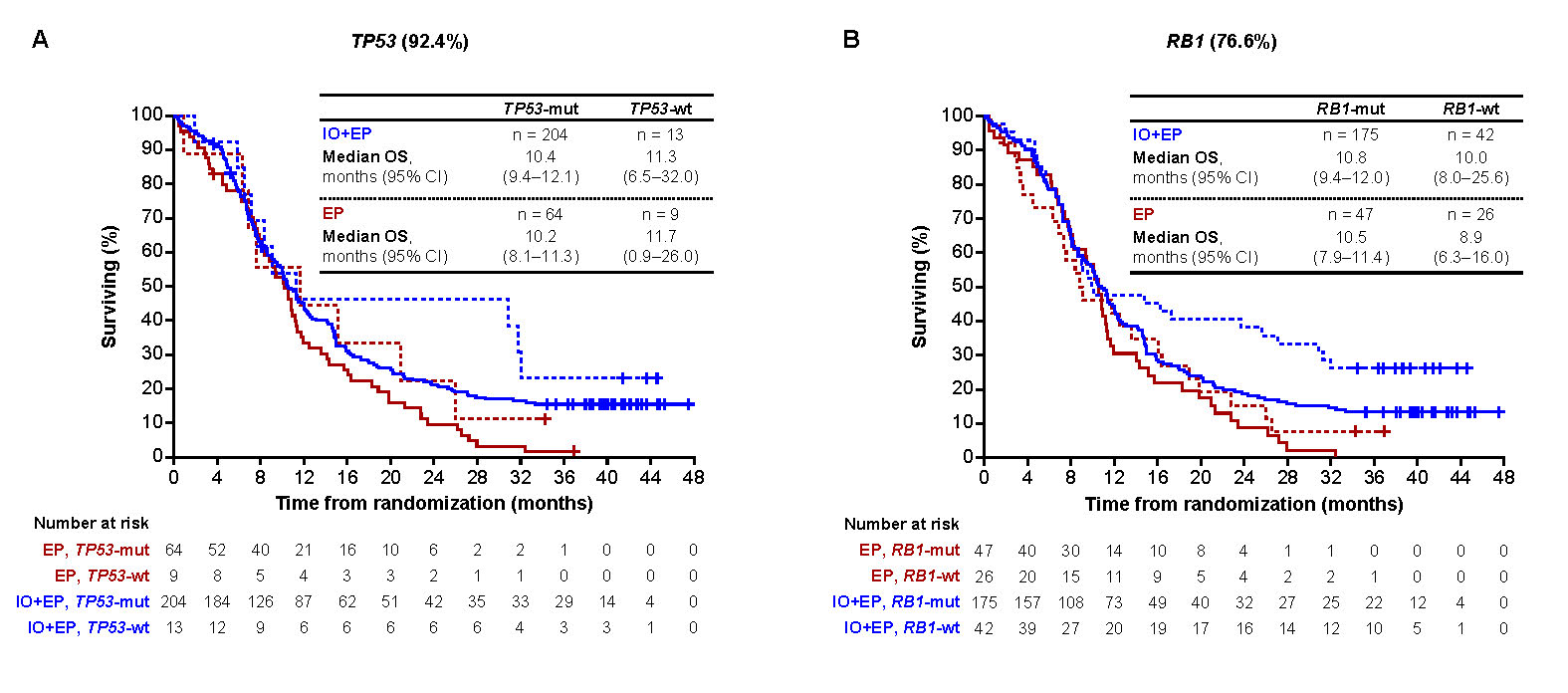


**Fig. S3** Tumor mutational burden in tumor tissue samples in the CASPIAN study in ES-SCLC and the MYSTIC and NEPTUNE studies in metastatic NSCLC. tTMB (mutations per megabase) was comparable across all three studies, with similar mean tTMB and no significant differences between any pairs of studies. tTMB distributions were compared between studies using t-tests.


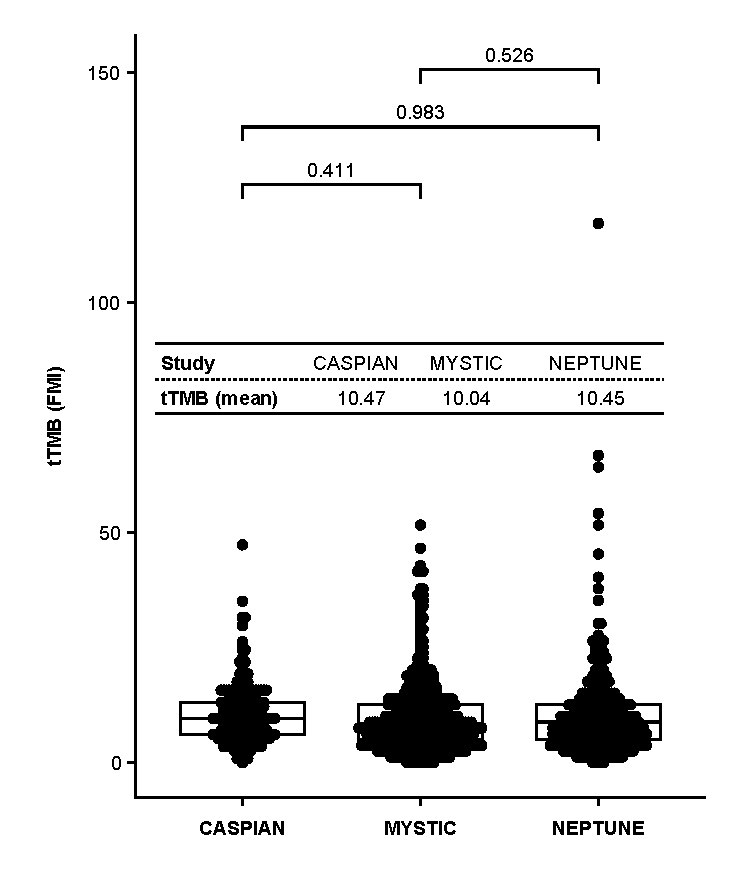


**Fig. S4** Immune phenotype and molecular subtyping, and association with OS in patients receiving immunotherapy plus EP or EP alone in CASPIAN. (A) Patients were grouped by PD-L1 expression of ≥1% or <1% on TC and/or IC, and categorized according to SCLC molecular subtype per the method of Rudin et al [5]. (B) T-cell inflamed signature in patient subsets defined by highest relative expression of single transcription factors (*ASCL1, NEUROD1, POU2F3, YAP1*). (C) Analysis of OS in patients treated with immunotherapy (IO; D±T) plus EP according to SCLC molecular subtype per the method of Rudin et al [5], and median OS by subtype and treatment received.


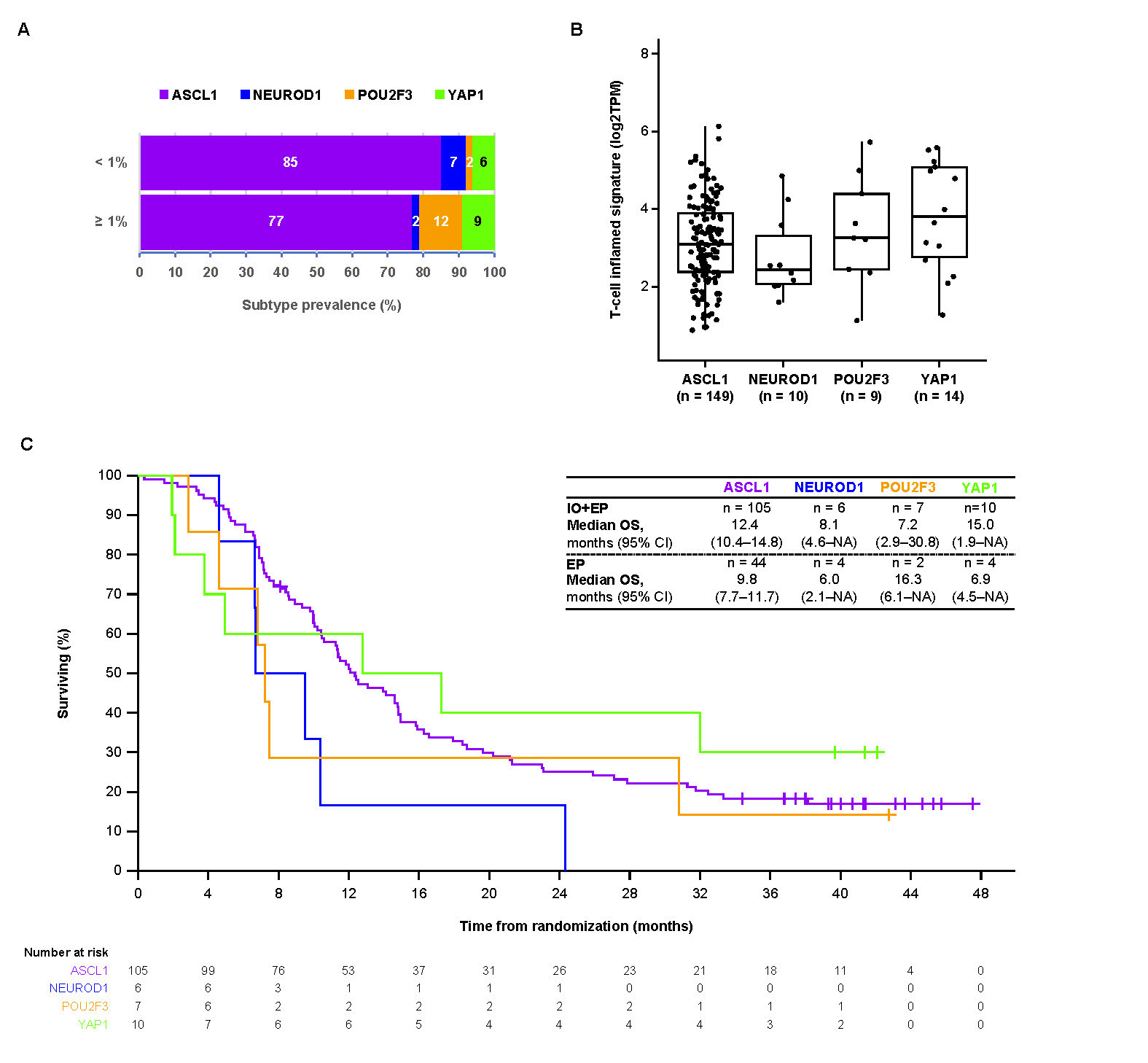


**Fig. S5** Gene expression profiling (RNAseq BEP) by OS. Upregulated/downregulated genes in patients receiving D+T+EP, D+EP, or EP alone who had OS of <18 or ≥18 months.


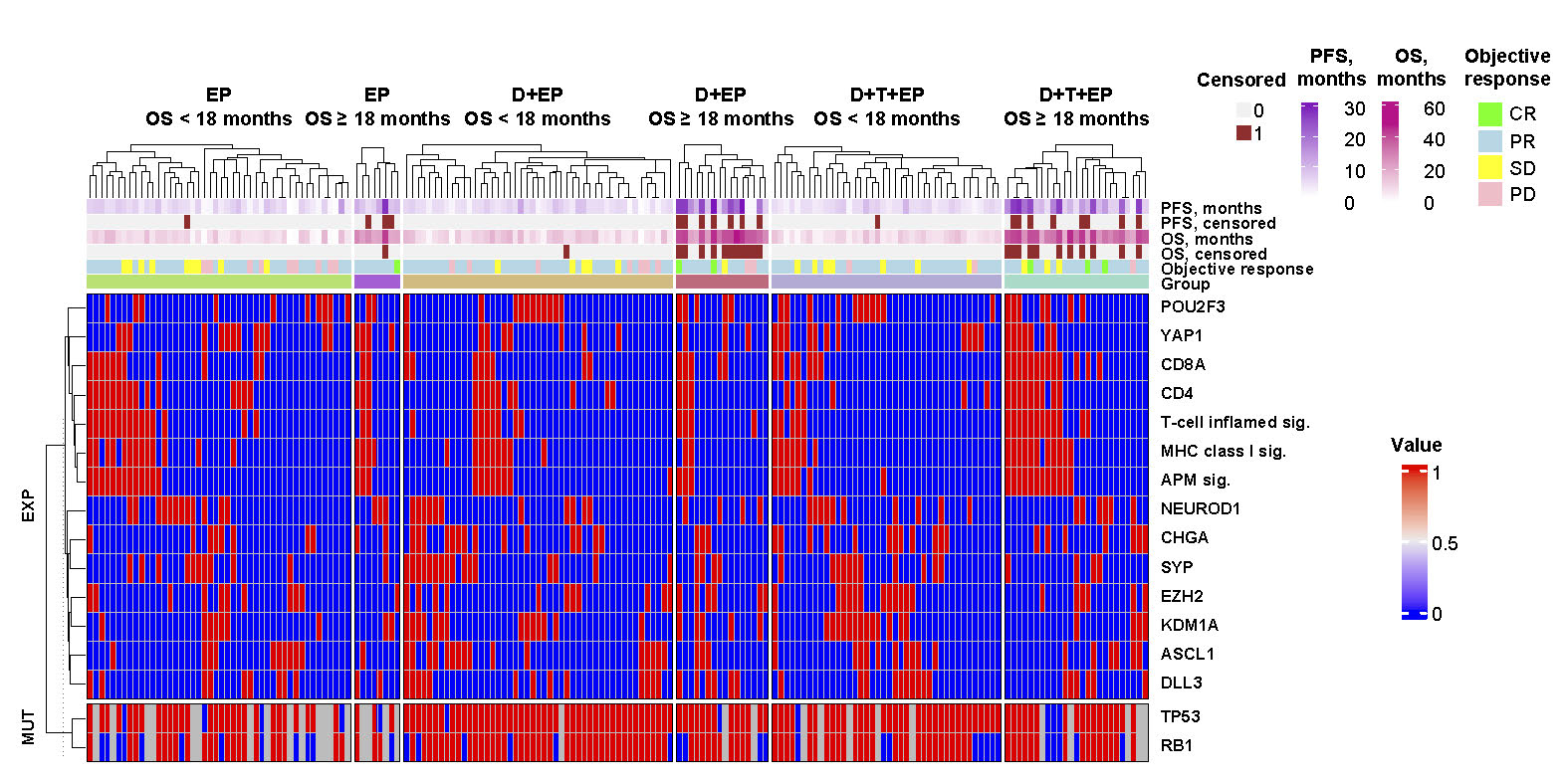


**Fig. S6** Markers related to immunotherapy benefit and CTLA-4 biology, and association with OS. OS with D+T+EP (left), D+EP (center), and EP (right) in patients with high (top quartile) vs low (other quartiles) (A) T-cell inflamed signature score, (B) *CD8A*, (C) *CD4*, (D) *CTLA-4*, and (E) *FOXP3* expression in the RNAseq BEP (n=182).


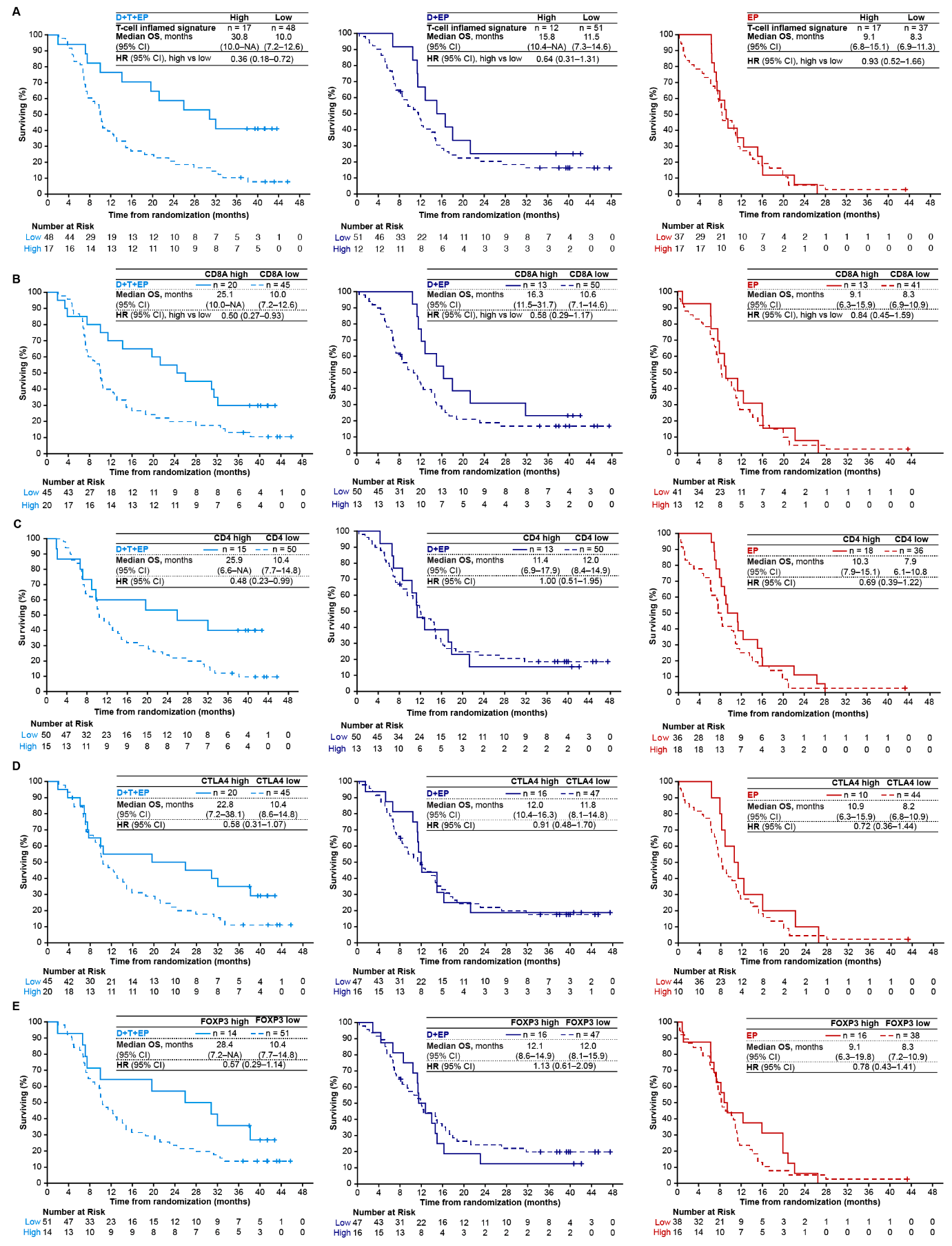


**Fig. S7** Gene set enrichment analysis of APM signature [MSigDB, KEGG_ANTIGEN_PROCESSING_AND_PRESENTATION] by treatment arm in CASPIAN. (A) D+T+EP, (B) D+EP, and (C) EP.


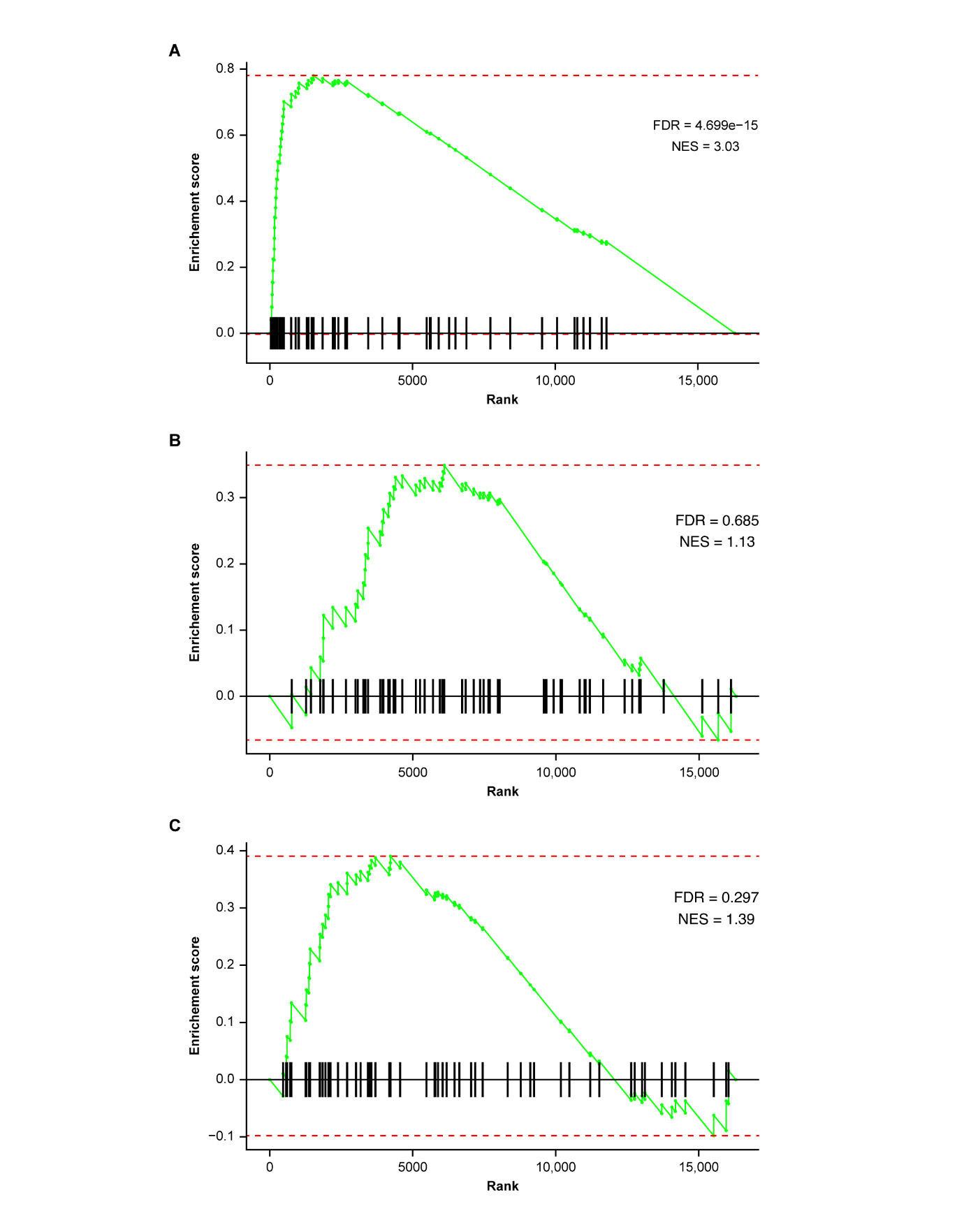


**Fig. S8** APM signatures by gene expression profiling (RNAseq BEP). Expression of (A) APM and (B) MHC I and II gene signatures according to SCLC molecular subtype per the method of Rudin et al [5].


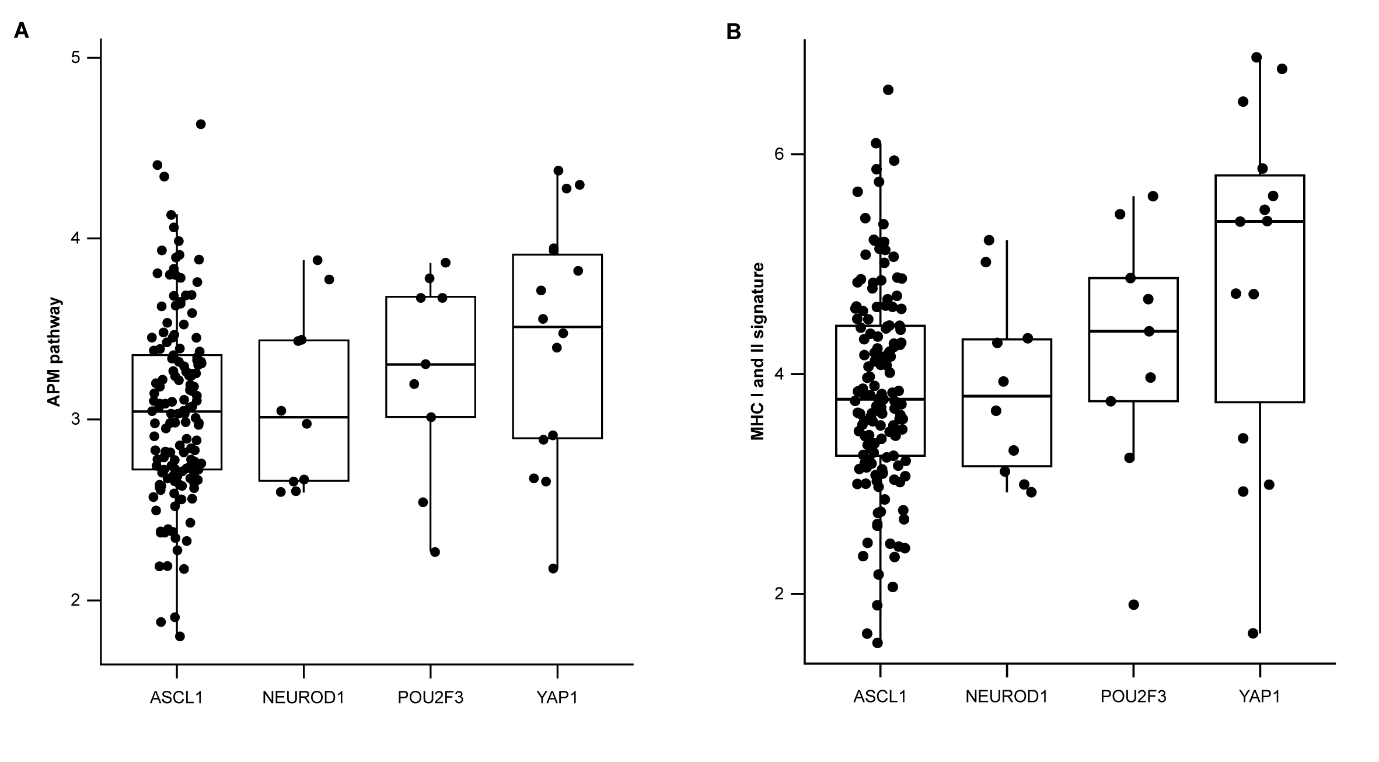


**Plain language summary**

Small-cell lung cancer (SCLC) is a fast-growing form of lung cancer that is named for how the cancer cells look when viewed under a microscope. Once SCLC spreads outside of a single area of the body it is known as extensive-stage SCLC (ES-SCLC). The first treatment for patients with ES-SCLC is a combination of chemotherapy and an ‘immunotherapy’ treatment, such as durvalumab. Durvalumab targets the immune system to help the body fight cancer by blocking the activity of a protein on cancer cells called PD-L1, making them more susceptible to being killed by immune cells.

Unfortunately, only a minority of patients respond to this treatment for a prolonged period of time. It is important to understand how ES-SCLC differs between groups of patients to see if there are any properties of the cancer that may help doctors understand who will respond to treatment. It has been suggested that patients with ES-SCLC can be classified into 4 subtypes based on how the expression of certain genes differs between patients – a subtype characterized by the expression of immunologically active genes (SCLC-I) has been observed to respond better to therapies that target the immune system to kill cancer cells.

CASPIAN was a clinical study in which participants with ES-SCLC who had not yet received any treatment were randomly assigned to treatment with etoposide and platinum-based chemotherapy (EP) alone, EP plus durvalumab, or EP plus durvalumab and tremelimumab. Tremelimumab is another ‘immunotherapy’ – an anti-cancer treatment that helps the immune system kill cancer cells, in this case by blocking the activity of a protein CTLA-4. Both the researchers and patients involved in CASPIAN knew which treatment was being given. Tumor samples were collected from patients and examined to see if specific genetic mutations or proteins were present in those patients who had prolonged survival with the addition of durvalumab, or durvalumab and tremelimumab, to their chemotherapy.

In this study, the most frequently observed mutations in ES-SCLC tumors were in the *TP53* and *RB1* genes, but neither of these mutations was associated with improved outcomes with durvalumab (±tremelimumab) when added to EP. The total number of mutations found in tumors was also not linked to improvements in outcomes with the addition of durvalumab (±tremelimumab). Consistent with what was already known, treatment with durvalumab (±tremelimumab) plus EP seemed to improve survival by the longest time in patients with the SCLC-I subtype. However, classifying patients by subtype alone was insufficient to identify those who had long-term benefit from treatment.

Patients whose tumors had the greatest density of cytotoxic T cells, a type of immune cell that can kill cancer cells, had longer survival with durvalumab (±tremelimumab) plus EP versus EP alone compared to patients whose tumors had a lower density of cytotoxic T cells (which were identified by a protein called CD8 on their surface or via expression of the *CD8A* gene). However, the density of cytotoxic T cells did not show which patients benefited from receiving tremelimumab in addition to durvalumab plus EP.

Instead, survival was improved by adding tremelimumab to durvalumab plus EP in patients whose T cells had a high expression of the gene *CD4*, and in patients whose cancer cells expressed genes that promote the creation of proteins on the surface of cancer cells, known as ‘antigens’, that allow binding to T cells. These findings suggest that there may be a subgroup of patients whose immune system within the tumor is ready to act and benefit from treatments – such as durvalumab and tremelimumab – that make cancers more susceptible to being killed by immune cells.

The findings of this study are therefore important because for the first time they provide evidence from a large group of patients with ES-SCLC that durvalumab and tremelimumab work with the immune system in the expected ways to kill cancer cells. The results also show that patients with particular ‘markers’ of these mechanisms are more likely to benefit from receiving these immunotherapies. Specifically, the findings suggest that measuring the expression of *CD8A* or the density of cytotoxic T cells with CD8 on their surface may help physicians identify those patients likely to benefit most from adding durvalumab to their chemotherapy. Similarly, the findings suggest that measuring the expression of *CD4* and the genes responsible for generating antigens may help physicians identify patients who might benefit from adding tremelimumab to durvalumab plus chemotherapy.
